# Detecting Early Cognitive Decline in Alzheimer’s Disease with Brain Synaptic Structural and Functional Evaluation

**DOI:** 10.3390/biomedicines11020355

**Published:** 2023-01-26

**Authors:** Samo Ribarič

**Affiliations:** Faculty of Medicine, Institute of Pathophysiology, University of Ljubljana, Zaloška 4, SI-1000 Ljubljana, Slovenia; samo.ribaric@mf.uni-lj.si

**Keywords:** Alzheimer’s disease, ATN biomarkers, cognitive decline, functional connectivity, hippocampus, large-scale networks, memory, structural connectivity, synaptic density, synaptic function

## Abstract

Early cognitive decline in patients with Alzheimer’s (AD) is associated with quantifiable structural and functional connectivity changes in the brain. AD dysregulation of Aβ and tau metabolism progressively disrupt normal synaptic function, leading to loss of synapses, decreased hippocampal synaptic density and early hippocampal atrophy. Advances in brain imaging techniques in living patients have enabled the transition from clinical signs and symptoms-based AD diagnosis to biomarkers-based diagnosis, with functional brain imaging techniques, quantitative EEG, and body fluids sampling. The hippocampus has a central role in semantic and episodic memory processing. This cognitive function is critically dependent on normal intrahippocampal connections and normal hippocampal functional connectivity with many cortical regions, including the perirhinal and the entorhinal cortex, parahippocampal cortex, association regions in the temporal and parietal lobes, and prefrontal cortex. Therefore, decreased hippocampal synaptic density is reflected in the altered functional connectivity of intrinsic brain networks (aka large-scale networks), including the parietal memory, default mode, and salience networks. This narrative review discusses recent critical issues related to detecting AD-associated early cognitive decline with brain synaptic structural and functional markers in high-risk or neuropsychologically diagnosed patients with subjective cognitive impairment or mild cognitive impairment.

## 1. Introduction

Alzheimer’s disease (AD) is a significant global public health challenge. The number of people with dementia is estimated to increase from 57.4 million to 152.8 million cases in 2050, and AD contributes to 60–80% of these cases. An early diagnosis can mitigate AD’s physical, psychological, social, and economic impacts; it enables the patient and carers to plan and implement lifestyle changes that slow the progression of AD and provide the patient with the best possible quality of life during the advancement of the disease [1,2].

AD is a protein-misfolding disease, a type of amyloidosis wherein, under incompletely understood conditions, specific proteolytic cleavage of APP initiates the production of protease-resistant, toxic amyloid β peptides (Aβ) with a β-sheet structure. A combination of several factors, including increased production, decreased clearance, and oxidative modification of these Aβ, promotes their aggregation into toxic oligomers, pre-fibrillar, fibrillar forms and amyloid plaques. This aggregation process is typical of all amyloidoses. It is dynamic, not unidirectional, since monomers and oligomers are continuously exchanged for each other depending on the physicochemical conditions of their environment. Successful disease-modifying therapies significantly reduced the tissue content of amyloid fibril masses in some patients. More than 30 types of amyloidosis were identified on the basis of their specific protein misfolding. Most amyloidoses are due to protein-misfolding of secreted proteins and the formation of extracellular protein deposits. Amyloidoses can affect several organs, for example, transthyretin amyloidosis, or are limited to a single organ, such as the brain in AD. Tissue damage in amyloidoses was first assumed to be due primarily to non-soluble amyloid deposits. Recently accumulated experimental and clinical evidence also implicates non-fibrillar oligomers in amyloidosis-associated tissue damage, for example, in AD-associated brain damage [3].

AD is a dual mixed proteinopathy with amyloid plaques, neurofibrillary tangles (NFT) and brain tissue atrophy, starting in the entorhinal region and the temporal lobe and progressing to the limbic system and the neocortex [4]. The associated biochemical, neurophysiological, and neuroanatomical changes can be measured decades before the clinically noticeable deterioration in cognition and behaviour [5]. In most patients, the initiating step for AD is not known. The number and extent of brain amyloid plaques have a weak correlation with the degree of cognitive decline. The absence of a robust correlation between Aβ plaques and cognitive impairment in patients with AD can be due to several non-excluding explanations, such as (a) person-to-person differences in the ability of inflammatory cells to remove senile plaques from the brain effectively; (b) the high neurotoxic properties of Aβ42 oligomers; and (c) person-to-person variations in the ability to sustain brain function in the presence of progressive brain injury [5].

Most patients with AD have a sporadic, late-onset form, where the major risk factors are ageing, type 2 diabetes (T2D) and apolipoprotein-E4 (APOEε-4). Identified LOAD genetic risk factors are APOEε-4 and mutations of TREM2, ADAM10 and PLD3 genes. A minority of AD patients have an early onset, genetic, familial form of AD due to autosomal dominant mutations in amyloid precursor protein (APP), presenilin-1 (PS1), and presenilin-2 (PS2) [4]. In addition to AD, other common causes of dementia are vascular dementia, Lewy body dementia, Parkinson’s disease with dementia and frontotemporal lobar degeneration. AD-associated brain changes coexist with vascular dementia. For example, brain blood vessels with amyloid wall deposition, endothelial cell degeneration, and reduction or obliteration of blood vessel lumen are associated with local neuropil and neuronal degeneration and astrogliosis, suggesting that perivascular cells and perivascular microglia contribute to amyloid fibril formation [6]. Therefore, comorbidities contribute to the variety of AD clinical signs and symptoms [4]. AD dementia is clinically diagnosed, since 20–40% of individuals aged 70 or above do not have cognitive impairment in the presence of biomarkers for AD or autopsy evidence of AD pathology [7].

Current AD drug therapy is symptomatic. Normal brain synaptic transmission is supported by NMDAR antagonists that attenuate excessive glutamate release and the related nerve cell death rate and cholinesterase inhibitors to conserve acetylcholine synaptic concentration. [8,9]. Recently, a double-blind, phase 3 trial evaluated the efficacy of a humanised IgG1 monoclonal antibody Lecanemab in persons with early AD. Lecanemab binds with high affinity to Aβ-soluble protofibrils. Compared to the placebo, 18 months of treatment with this antibody reduced amyloid markers on PET brain scans and attenuated cognitive decline, but was associated with adverse events. The authors suggest longer trials to determine the efficacy and safety of Lecanemab in treating early AD [10]. AD is a multifactorial disease, and the current treatment approach is shifting from a single pathological target towards developing a stage-specific strategy for a comprehensive and personalised treatment of patients with AD that combines pharmacological and non-pharmacological treatment interventions [8].

Cognitive decline is a hallmark of AD and is associated with structural and functional connectivity changes in brain networks. The building blocks of these networks are neurons that communicate by chemical or electrical synapses and glia, which support neuronal function and modulate the interneuronal transmission of action potentials. This narrative review discusses recent critical issues related to detecting AD-associated early cognitive decline with brain synaptic structural and functional markers in high-risk or neuropsychologically diagnosed patients with subjective cognitive impairment or mild cognitive impairment.

### 1.1. Brain Synapses

Neurons and glia (i.e., neuroglial cells including astrocytes, oligodendrocytes, ependymal cells, and scavenger cells—microglia) represent the brain’s functional tissue. Neuroglial cells provide nutrients to neurons, and astrocytes also modulate interneuronal communication. Neurons are the primary electrically excitable cells, and form neural circuits with other neurons over which action potentials and nerve impulses are conducted [11].

The site of communication and transmission of an action potential among brain nerve cells is the synapse, formed by discrete, specialised plasma membrane sections between connecting neurons. The number of synapses per brain nerve cell depends on the nerve cell type and numbers in the thousands [12]. Brain synapses are classified by the:

- location of synapses,
- electrical or chemical mode of transmission,
- type of synaptic neurotransmitter, excitatory or inhibitory [13], and
- interneuronal protein transport [14].

The number of brain synapses is not constant from birth and reflects the balance between synaptogenesis (present during the embryonic, neonatal, and adolescent life period) and synaptic pruning, pronounced during adulthood when synaptogenesis is very limited. The numbers of neurons and their synapses in the human adult brain are colossal. The estimated number of neurons is 86 × 10^12^, and the number of non-neuronal cells is about 85 × 10^12^. The estimated percentage of neurons in the cerebral cortex (the binding site of neural integration in the brain) is 19% [15]. The cumulative number of synapses in the neocortex (the central region of the cerebral cortex, essential for cognition and other higher-order brain functions) was estimated at 164 × 10^12^ with an average, rounded-up number of 7 × 10^3^ synapses per neuron [16]. The overall effect of a large number of synaptic connections per neuron is an exponential increase in cognitive abilities with the absolute number of neurons integrated into brain neuronal circuits [15].

Synaptic density refers to the number of brain synapses during adulthood that, in the absence of neurodevelopmental or neurodegenerative disorders, does not change significantly over time and thus enables the detection of brain disorders with a reduced number of synapses [11]. For example, a reduced cortical and hippocampal synaptic density in patients with AD is associated with degraded memory, attention and thought organisation [11,17,18]. Additionally, synaptic loss changes functional neuronal connectivity among brain regions, which underpins the measured cognitive decline (e.g., mini mental status examination) in patients with AD (discussed in Section 3.3).

#### 1.1.1. Brain Chemical Synapses

**Figure 1:**
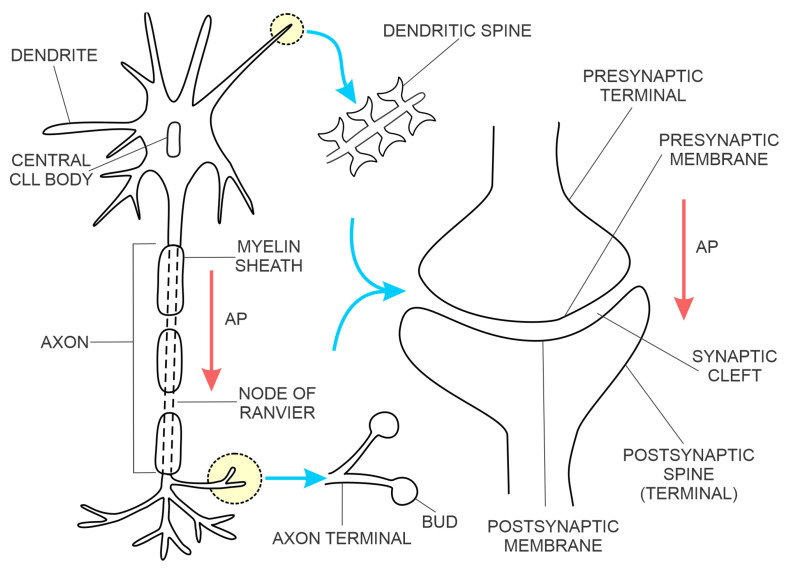
Nerve cell. Abbreviation: (AP with red arrows) direction of action potential transmission.

The most common type of brain synapse releases a chemical neurotransmitter from the presynaptic membrane of the upstream neuron into the synaptic cleft, where it diffuses and binds to a receptor on the postsynaptic membrane of the downstream neuron, thus increasing or decreasing the probability of action potential. In addition to the number of activated excitatory synapses per unit of time, their dendritic spatial proximity on the downstream neuron also determines the probability of action potential [12]. The synaptic contact points of the upstream neuron are projections of the axon terminals, forming the presynaptic membranes (Figure 1). The synaptic contact points of the downstream neuron are dendritic spines (protrusions along the dendrites) created by the postsynaptic membranes [19]. The postsynaptic membranes have three classes of proteins:

- G-protein-coupled transmembrane receptors (e.g., glutamate, muscarinic acetylcholine and gamma-aminobutyric acid B (GABAB) receptors) coupled to intracellular metabolic pathways;
- ligand-gated ion channels, also known as ionotropic receptors (e.g., glutamate receptors (α-amino-3-hydroxy-5-methyl-4-isoxazole propionic acid—(AMPA)) and the N-methyl-D-aspartate receptor, a tetrameric protein with two GluN1 subunits and two GluN2 (A-D) subunits; a type of L-glutamate (NMDA) transmembrane ion-channel protein that enable ions (e.g., Na^+^, K^+^, Ca^2+^) to cross through the cell membrane in response to binding of a ligand (e.g., neurotransmitter); and
- scaffolding proteins, the postsynaptic density (PSD) proteins that anchor the transmembrane receptors [20].

**Figure 2:**
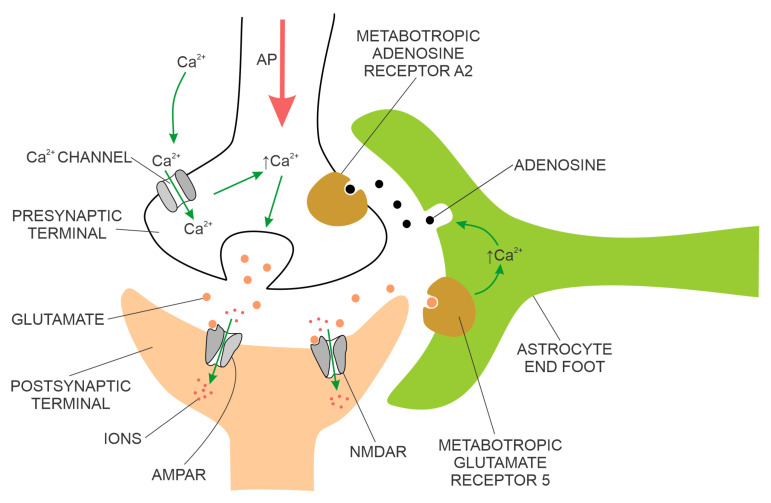
The tripartite synapse. Abbreviations: AMPAR (α-amino-3-hydroxy-5-methyl-4-isoxazolepropionic acid receptor), NMDAR (N-methyl-D-aspartate receptor, tetrameric protein with two GluN1 subunits and two GluN2 (A-D) subunits, a type of L-glutamate receptor). The AP with red arrow represents the direction of action potential transmission.

The physical proximity and functional integration of the presynaptic and postsynaptic membranes of the chemical synapse on the one hand and the astrocyte on the other are collectively named the tripartite synapse (Figure 2). These synapses are located throughout the human brain, including the hippocampus. It is estimated that a single astrocyte is in contact with 27 × 10^4^ to 2 × 10^6^ synapses in the human brain [21]. Astrocytes in tripartite synapses are vital for neurons; in the absence of astrocytes, cortical synapses cannot develop, and developed neurons cannot survive. In addition to supporting neuronal viability and synaptic morphology, astrocytes also modulate the transmission of chemical synapses through bidirectional communication between astrocytes and neurons. For example, the conduction of the action potential across the chemical synapse elicits a transient increase in intracellular Ca^2+^ concentration from the astrocyte and the release of astrocytic factors that modulate synaptic plasticity in a feedback manner. An example of an astrocytic growth factor is hevin, which promotes post-synaptic membranes rich in NMDARs and low in AMPARs additionally, the expression of post-synaptic proteins (e.g., PSD95, Homer 1, NMDAR subunits GluN1 and GluN2B, and AMPAR subunit GluA2—this AMPAR subunit prevents Ca^2+^ flux through AMPAR) is increased. The astrocyte’s response to synaptic transmission across the pre-and post-synaptic membranes can be chemical transmitter specific, e.g., responding selectively to either acetylcholine or glutamate [22,23].

Interneuronal protein transport is present in the normal brain (e.g., the transport of brain-derived neurotrophic factor (BDNF)) and in neurodegenerative brain disorders, e.g., the transport of toxic forms of amyloid, tau and αα-synuclein. In animal models of AD, where aggregation of amyloid β (Aβ) peptides to Aβ plaques was elicited by the application of synthetic Aβ fibrils or brain extracts from patients with AD, the pattern of Aβ plaques’ distribution followed the neuronal pathways between the site of injection and the affected distant brain regions [24]. However, the extent of interneuronal protein in an adult, healthy brain has not been quantified until recently. In 2022, a study of trans-neuronal protein transport in the brain of normal adult rats identified about 200 proteins, transported mainly in neuronal exosomes between neurons of the rodent’s visual cortex. These proteins included a large majority of synaptic proteins. The observed trans-neuronal transport of these proteins was preferential to excitatory neurons [14]. Exosome-transported tau oligomers were identified in the postsynaptic neurons [25,26].

#### 1.1.2. Brain Electrical Synapses

**Figure 3:**
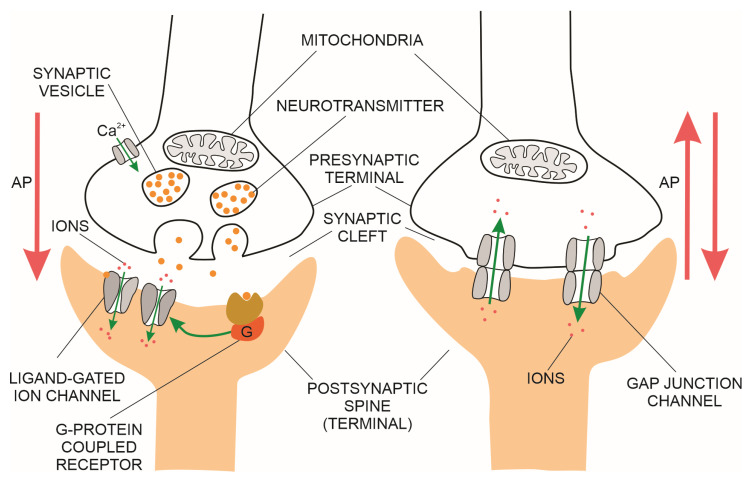
Chemical synapse (left) and electrical synapse (right). The AP with red arrows represents the direction of action potential transmission.

An electrical synapse is constituted of neuronal gap junction channels that span the presynaptic and postsynaptic membranes (Figure 3 right side), directly connecting the intracellular spaces of the two neurons and enabling the spread of electrical currents between them [27]. In the mammalian brain, the electrical synapses are widely distributed. However, they are less numerous compared to chemical synapses. Both electrical and chemical synapses have activity-dependent plasticity and potentiation of either electrical or chemical transmission blocked by NMDAR antagonists [28,29]. Modelling of fast-spiking interneurons’ activity at gamma frequency in networks with strong chemical and weak electrical coupling supports the hypothesis that chemical and electrical synapses perform complementary roles in the synchronisation of interneuronal brain networks [30].

There are three types of electrical synapses, classified by the location of neuronal gap junction channels:

- distal dendro-dendritic (most common in mammals);
- somato-somatic;
- between axons (essential for high-frequency neuronal circuits synchronisation in mammals), and
- mixed chemical-electrical synapses with glutamate neurotransmitters [31].

Transmission over electrical synapses is regulated by the number of gap junction channels and is continuous. There is no synaptic cleft as in chemical synapses, and no synaptic delay. These synapses have a bidirectional transmission; the coupling potential in the post-synaptic cell (elicited by the action potential in the pre-synaptic cell) is also concurrently transmitted back to the presynaptic cell, provided there are no voltage-gated ion channels that open in response to depolarisation of an axon’s plasma membrane and attenuate the retrograde current [31,32]. Synaptic transmission of electrical synapses depends on the conductance of the gap junctions, the presynaptic signal’s duration, and the postsynaptic neuron’s time constant. Therefore, individual action potentials, long-term depolarisations or long-term hyperpolarisations will each trigger different net postsynaptic average voltage or current changes between coupled neurons [31]. Neuromodulators of gap junction channels (e.g., dopamine) are released from pre-synaptic terminals of chemical synapses. They bind to metabotropic G-protein-coupled receptors, thus triggering signalling pathways that modulate connexin and innexin gap junction proteins [33,34]. In contrast to chemical synapses, which have excitatory or inhibitory effects on the post-synaptic neuron, electrical synapses can excite or inhibit their coupled neurons [35].

In mammals, dendro-dendritic gap junction channels contribute to oscillatory brain activity. For example, the generation of gamma-frequencies in the neocortex, thalamus and hippocampus is associated with cognitive processing (e.g., associative binding, learning, and memory) and was attributed to the high-frequency activity of cortical inhibitory interneurons with electrical synapses [36,37,38,39,40,41]. The bi-directionality of synaptic transmission, in conjunction with the specific location of gap junction channels, enables the modulation of electrical communication in neuronal circuits. For example, electrical synaptic transmission facilitates synchronisation (over axo-axonal synapses) among large groups of neurons connected in neural circuits that are subjected to simultaneous subthreshold depolarisations [32,42,43,44,45,46], and distal dendro-dendritic electrical synapses can promote lateral excitation and increased efficiency of mammalian escape neuronal circuits [32].

The unique properties of electrical synapses in the mammalian brain provide an additional modality to increase the adaptability of neuronal pathways with chemical neurotransmitters [31,32,47]. Electrical coupling between GABAergic interneurons underpins their synchronous activity and sustains the synchronised activity in neocortical pyramidal cells [36,48,49,50,51,52,53]. Hippocampal electrical synapses facilitate synchronisation among inhibitory interneurons, which is also associated with high-frequency γ-oscillations in pyramidal cells [37,54,55,56,57,58,59].

### 1.2. Brain Neuronal Circuits

Brain neurons communicate directly with adjacent neurons through synapses and indirectly with many neurons connected in neuronal circuits. The architecture of brain neuronal circuits was comprehensively summarised by Luo (2021) into two classes: (a) basic neuronal circuits and (b) function-specific, specialised circuits [60]. Except for biased input–segregated output circuits, all neuronal circuits are built from excitatory and inhibitory neurons. Most examples of neuronal circuits were identified in the mammalian visual system. Basic neuronal circuit structures include feedforward excitation, feedforward/feedback inhibition, lateral inhibition, and mutual inhibition. Examples of mammalian reciprocal inhibition circuits that exhibit rhythmic activity on either short or long-time scales are:

- locomotion circuits;
- neocortical circuits with three types of inhibitory neurons that have their site-specific pre-synaptic contacts with the pyramidal neurons’ postsynaptic dendrites, central cell bodies and proximal axonal segments, respectively; and
- sleep–wake cycle neuronal circuits.

Examples of function-specific, specialised neuronal architectures are:

- continuous topographic mapping (i.e., transmitting and storing spatial information from the outside world or human body parts in topographic maps stored in specialised cortical regions);
- discrete parallel processing (i.e., increasing information processing speed),
- dimensionality expansion;
- recurrent loops, where nerve cells connect back onto themselves with intermediary neurons; and
- biased input–segregated output neuronal circuits.

The speed of discrete parallel information processing in the human brain depends on the number of participating neurons and synapses. Discrete parallel processing and continuous topographic mapping neuronal circuits coexist in the visual pathway and enable parallel processing of different image qualities, i.e., luminescence, motion and colour. Dimensionality expansion neuronal circuits enable the “learning” of output neurons by adjusting their synaptic plasticity with a relatively small number of input “teaching” neurons, for example, in the cerebellum. The hippocampal neuronal circuits are assumed to be self-learning, since no “teaching” neurons have been identified to date [60]. Biased input–segregated output neuronal circuits have neurons with monoamines or neuropeptides neurotransmitters (released into the synaptic cleft or outside the synapses) that bind to G-protein-coupled receptors on the postsynaptic membrane to elicit slower and longer-lasting responses (up to 1 s) compared to fast excitatory or inhibitory neurotransmitters. In mammals, neurons from these circuits receive inputs from neurons in similar regions (external and internal stimuli regulate these inputs). However, each subgroup has segregated output projections to discrete brain regions that serve specific behavioural functions [60].

Brain neuronal circuits interconnect and create large-scale brain circuits wherein widespread brain regions coordinate their electrical activity in time (i.e., they have functional connectivity, the strength of which is evaluated by the functional magnetic resonance imaging (fMRI) blood oxygenation level-dependent (BOLD) signal correlation among brain areas). The level of functional connectivity among brain regions is detected and assessed by statistical analysis (e.g., cluster analysis and spatial independent component analysis) of fMRI, quantitative-electroencephalography (EEG) or positron emission tomography (PET) records. Due to the brain’s complexity, derived from the large numbers of neurons and synapses, large-scale brain neuronal circuits can only be represented as an approximation by mathematical models called neuronal networks. The quality of human cognition is assumed to be critically dependent on the appropriate activity pattern of brain neuronal circuits that connect several discrete brain regions [11].

### 1.3. Alzheimer’s Disease

AD is a chronic, neurodegenerative disorder with a survival rate of 3 to 9 years. Most patients with AD have the late-onset form (LOAD), where the signs of cognitive decline are preceded by ten or more years of disease development and progression without any clinical signs of cognitive impairment. Several risk factors for LOAD were identified, some of which are modifiable (e.g., high cholesterol, type II diabetes and high blood pressure) and can be managed to reduce the risk for LOAD or attenuate the progression of cognitive decline to dementia in LOAD. Ageing is the most critical risk factor for LOAD [61,62].

During the first presymptomatic stage, toxic Ab and tau aggregate products accumulate in the hippocampus, a brain region essential for memory and learning. The second, pre-dementia, symptomatic stages of AD are at first, subjective cognitive impairment, the self-experienced cognitive decline not measurable through formal neuropsychological testing (SCI), and later, mild cognitive impairment (MCI), where the loss of hippocampal neurons and reduced hippocampal synaptic density leads to a mild and measurable degradation of the short-term memory and other cognitive skills [61,63,64,65].

Patients with subjective cognitive impairment (SCI) or mild cognitive impairment (MCI) can still live independently and perform daily activities. AD is not the only cause of MCI. Other dementia-linked causes for MCI are neurodegenerative disorders (Parkinson’s disease, frontotemporal dementia, dementia with Lewy bodies, and vascular dementia). Causes for MCI not linked to neurodegenerative disorders are depression, anxiety, stress, vitamin or thyroid deficiencies and side effects of medication. AD and MCI share some modifiable (high cholesterol, type 2 diabetes, high blood pressure, obesity, depression, lack of physical exercise) and non-modifiable (increasing age) risk factors [61,62,66].

The second phase lasts 2 to 7 years before progressing to the final, third stage, with MRI-detectable brain cortex atrophy and disruption of white matter integrity. There is reduced functional connectivity in the multiple default mode network (DMN), accompanied by dementia (severely impaired short-term and long-term memory, language, cognitive and motor function), wherein the decline in cognitive function severely interferes with daily living. The third phase of AD lasts 3 to 8 years until death. Patients with AD represent the largest subgroup of all dementia patients (60%–80%) [61,62].

## 2. Synapse Structure and Function in Alzheimer’s Disease

### 2.1. Ageing

Typical ageing (the life interval between adulthood and old age) of the brain neurons is accompanied by a modest cognitive decline associated with reduced spine volume. The postsynaptic (dendritic) membranes are more affected by normal ageing than the presynaptic (axon terminal) ones. The more prominent spines are more resistant to ageing-related changes than the smaller ones [18]. There is a reduced variety in the shape and size of spines. However, the spine density does not change during normal ageing [67].

The tolerance of mammalian brain neurons to the toxic effects of soluble Aβ aggregates is enhanced by the glycoprotein reelin, released by cortical pyramidal cells. Reelin promotes NMDA activation and long-term potentiation (LTP) and attenuates NMDAR endocytosis by phosphorylating NMDAR’s GluN2 subunit. Ageing, chronic inflammation, and apolipoprotein E4 (ApoE4) reduce the availability of synaptic reelin receptors and attenuate reelin-elicited GluN2 subunit phosphorylation and reelin-mediated nerve cell resistance to the toxic effects of soluble Aβ accumulation, thus promoting the loss of brain synapses, chronic neuroinflammation and increased accumulation of toxic soluble Aβ aggregates near synapses [18].

### 2.2. Synapse Morphology and Proteins

In AD studies, synaptic loss and reduced plasticity preceded the death of brain neurons. This was confirmed in various cell cultures, whole animal, and human studies [18,20,68,69]. Synaptic loss, an early brain pathological change in AD most pronounced in the neocortex and hippocampus, is due to local synaptic accumulation of toxic soluble amyloid β oligomers (AβOs), phosphorylated tau and increased free radical production in mitochondria (MT). It is also an early cause of AD-related cognitive decline [18]. In addition to reduced synaptic density and plasticity, the accumulation of toxic Aβ aggregates also changes the shape and composition of synapses. The content of pre-and postsynaptic proteins is decreased in patients with AD; for example, there is a reduced density of presynaptic proteins synaptosomal-associated protein 25 (SNAP-25), synaptophysin, and synaptotagmin, and reduced content of structural protein drebrin located in dendritic spines [70,71]. In patients with AD, changes in synaptic protein content preceded Aβ plaque formation [18].

### 2.3. Amyloid β Processing

Brain amyloidosis per se is not always associated with dementia. In a cross-sectional study of 598 amyloid-positive participants identified by brain PET imaging, the highest CSF soluble (Aβ42) levels were associated with normal cognition, and average hippocampal volume, despite advanced brain amyloidosis detected on PET scans [72]. In general, brain amyloid-beta plaque deposition in animal and human models of AD is associated with synapse loss and memory deficits. In humans, the progression of Aβ plaques within the brain is divided into five phases, starting with plaque appearance in the neocortex and ending with plaque distribution in the cerebrum, cerebellum and brain stem in phase five [73]. The most strongly associated factor with cognitive deficit and synaptic loss are toxic soluble AβOs [17]. AβOs accumulate preferentially at postsynaptic and presynaptic terminals of excitatory synapses [74]. The critical step in toxic soluble AβOs’ formation are conformational changes of modified Aβ42 peptides into reactive, toxic metastable oligomers (i.e., the primary nucleation processes) that further assemble into protofibrils, fibrils and amyloid plaques. The secondary nucleation processes further accelerate the conversion of Aβ42 peptides to toxic AβOs at the AβO, protofibril and fibril nucleation sites [75,76].

In the short term, toxic soluble AβOs inhibit LTP and reduce the expression of synaptic proteins essential for normal neurotransmission, memory and learning and, in the long term, lead to the loss of spines and synapses in both animal models of AD and the human brain of patients with AD [77,78,79,80,81,82,83]. Toxic AβOs bind to several synaptic receptors (e.g., AMPA receptors, NMDA receptors, metabotropic glutamate receptor 5 (mGluR5) receptors, receptors for advanced glycation end products (RAGE), cellular prion protein, negative growth regulatory protein (NGR1NgR1), ephrin type-B receptor 2 (EphB2), and PirB/LilrB2), promoting:

- unregulated Ca^2+^ influx associated with increased oxidative stress and production of free radicals, both elicited by binding to mGluR5 and NMDA receptors;
- prolonged long-term depression (LTD) by binding with AMPA receptors that stimulate their internalisation;
- reduced levels of phospho-Akt with reduced expression of heat shock proteins;
- increased activation of glycogen synthase kinase-3β (GSK3β), leading to a further increased production of toxic AβOs;
- loss of dendritic spines, dendritic shrinkage and collapse, and
- apoptosis [84,85,86,87,88,89,90,91,92,93].

There is no statistically significant difference in toxic AβOs levels between early and late AD. It has been suggested that there is an oligomeric amyloid beta threshold in early stage AD, when the risk for AD-related dementia increases significantly [94].

### 2.4. Tau Processing

The second pathognomonic feature of AD, in addition to the accumulation of toxic Aβ products, is the aggregation of truncated, misfolded, and hyperphosphorylated tau into soluble and highly reactive and toxic oligomers. Untruncated forms of soluble tau oligomers further aggregate into insoluble neurofibrillary tangles (NFT). The transformation of tau to oligomers and NFT is associated with an early reduced dendritic spine size and synapse loss, and later with neuronal death and disruption of neuronal networks [17]. The transformation of normal tau (a hydrophobic, microtubule-associated protein that supports the integrity of neuronal microtubules and axonal transport) to toxic, truncated tau oligomers is promoted by AβO (via protein kinase A (PKA) and Ca2+/calmodulin-dependent protein kinase II (CaMKII) activation), caspases, increasing activity of cyclin-dependent kinase 5 (cdk5) and GSK3β. Reelin inhibits GSK3β and thus attenuates tau phosphorylation; however, reelin production decreases with brain ageing [18]. Truncated tau forms do not form NFT [95]. The distribution of neurofibrillary tangles (NFT) in the human brain is quantified by Braak staging, where NFT are initially localised to the transentorhinal region of the brain (stages I and II), later including the limbic areas (stages III and IV) and finally spreading to the neocortex in stages V and VI [96]. Cognitive decline due to tau pathology in AD is measurable in Braak stages III to VI [18].

**Figure 4:**
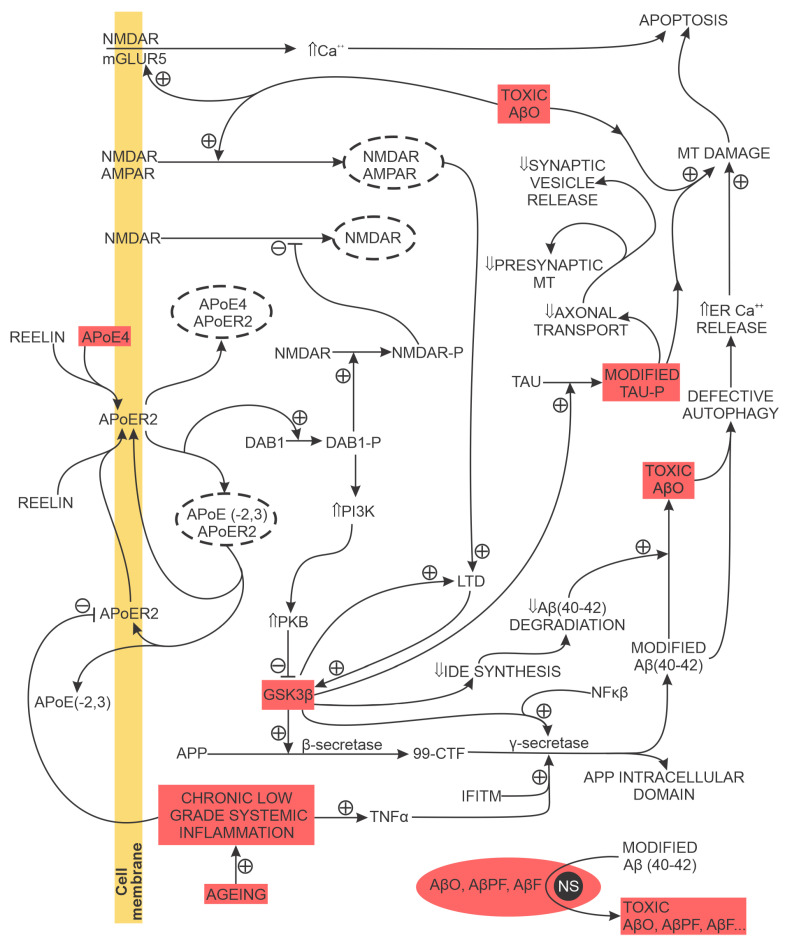
Toxic soluble AβO and truncated, misfolded, and hyperphosphorylated toxic tau oligomers are the critical drivers of synapse loss and neuronal death in AD. The transformation of normal tau to its toxic oligomers is promoted by increased GSK3β activity; GSK3β inhibits Aβ42 degradation and promotes Aβ42 production by stimulating β- and γ-secretase activity. Increased TNFα and IFITM levels also promote high GSK3β activity. Ageing-associated chronic low-grade sterile inflammation and reduced reelin synthesis facilitate Aβ metabolism dysregulation. The secondary nucleation processes further accelerate the conversion of Aβ42 peptides to toxic AβOs at the AβO, protofibril and fibril nucleation sites. Abbreviations: ⇑ (increased), ⇓ (decreased), + (stimulating effect), − (inhibitory effect), - - - - (dashed lines denote internalised molecules), 99-CTF (99-amino acid membrane-bound C-terminal fragment), Aβ42 (an amyloid β peptide with 42 amino acid residues), AβO (amyloid β oligomers), AMPAR (α-amino-3-hydroxy-5-methyl-4-isoxazolepropionic acid receptor), APP (amyloid precursor protein), AβF (amyloid β fibril), AβPF (amyloid β protofibril), APOE (apolipoprotein E), APOER2 (reelin apolipoprotein E receptor 2), DAB1 (DAB adaptor protein 1), DAB1-P (phosphorylated DAB adaptor protein 1), GSK3β (glycogen synthase kinase 3β), IDE (insulin-degrading enzyme), IFITM (interferon-induced transmembrane protein), mGLUR5 (metabotropic glutamate receptor 5), NFκB (nuclear factor kappa-light-chain-enhancer of activated B cells), NMDAR (N-methyl-D-aspartate receptor), NS (nucleation site), PI3K (phosphoinositide 3-kinase), PKB (protein kinase B), Tau-P (truncated, misfolded, and hyperphosphorylated tau), TNFα (tumour necrosis factor α).

AD-associated hyperphosphorylated tau disrupts the neuron’s microtubule-based cellular transport over which the neuronal synapses (on the dendritic-postsynaptic and an axon terminal (presynaptic) side) receive MT, glutamate receptor subunits for the postsynaptic membrane and other molecules essential for synapse maintenance and normal function. For example, a reduced synaptic MT content (a) reduces or blocks presynaptic vesicle release, probability for transmission of the action potential due to insufficient ATP and (b) prevents synaptic intracellular Ca^2+^ buffering. Ultimately, the hyperphosphorylated tau-elicited disruption of microtubule-based cellular transport leads to loss of synapses (Figure 4). Examples of the additional detrimental effects of modified tau are (a) the binding of modified tau to PSD-95/NMDA receptor complex, increasing glutamatergic-transmission-associated NMDA activation; (b) the loss of dendritic spines due to stimulated calcineurin activation and (c) the reduction of AMPAR in the postsynaptic membrane associated with learning and memory deficit and (d) the synaptic and Ca^2+^-dependent neuron-to-neuron transmission of tau [18].

In AD, the accumulation of synaptic Aβ products precedes phosphorylated tau. Additionally, the intracellular mislocalisation of phosphorylated tau is promoted by AβO. Experimental results support the existence of a tau-amyloid beta synergism that accelerates synapse loss, neuronal death, and cognitive impairment in AD. AβO increases tau concentration at the post-synaptic, dendritic sites. This locally increased tau concentration is assumed to concentrate proto-oncogene tyrosine-protein kinase Fyn (FYN), increase NMDA receptor activity, and increase Ca^2+^ influx with Ca^2+^ dyshomeostasis. In addition, AβO directly promotes the transformation of normal tau to hyperphosphorylated tau and, indirectly, to later toxic, truncated tau oligomers by reducing tau ubiquitin-ligase-mediated degradation of hyperphosphorylated tau [18,97,98].

### 2.5. Mitochondrial Energy Production

The primary site of MT synthesis is the neuronal central cell body (i.e., soma). MT must be transported to distal energy-intensive sites, to synapses concentrated at the axon terminals and dendrites, to provide sufficient ATP for energy-intensive synaptic activities (synaptic growth, synaptic vesicle formation and synaptic transmission). Therefore, damage to neuronal MT synthesis in the soma (due to ageing, toxic AβO, or toxic phosphorylated tau oligomers) and to the anterograde axonal transport will be reflected in a reduced ATP supply at the dendritic and axon terminal synapses, degrading synaptic vesicle formation, transmission, Ca^2+^ transport and outgrowth [18,99,100].

Toxic AβOs damage MT located in the soma and at the synaptic terminals. They bind to MT fission, outer-membrane, and matrix proteins, eliciting increased production of free radicals, enhanced MT fragmentation, reduced adenosine triphosphate (ATP) production, and loss of MT Ca^2+^ intracellular homeostasis with increased intracellular Ca^2+^ that promotes unregulated neurotransmitter release from the presynaptic terminal, and ultimately, apoptosis. Mitochondrial deoxyribonucleic acid (DNA) is highly susceptible to oxidative stress due to its proximity to the site of reactive oxygen species (ROS) generation and lack of histones or other alternative DNA-repairing mechanisms. In AD, toxic AβOs bind to VDAC1 (bi-directional, voltage-dependent, anion transport channels in the outer MT membrane), leading to defective oxidative phosphorylation, increased MT ROS production, MT DNA damage, MT fragmentation, reduced clearance of damaged MT and, ultimately, apoptosis [18]. Studies on animal models of AD linked abnormal MT changes to synaptic dysfunction and learning and memory impairment [101,102,103,104].

### 2.6. Microglial Activation

Ageing is associated with increased brain levels of pro-inflammatory cytokines (e.g., TNF α) and increased brain aggregation of pro-inflammatory (M1) microglia immune cells. In AD, toxic AβO converts, in a concentration-dependent manner, M2 (anti-inflammatory) microglia immune cells to a unique subtype of abnormal M1 microglia immune cells not found in the normal brain. This AD-associated subtype of M1 cells overstimulates AMPA receptor activity and promotes the loss of synapses at the neurons’ dendritic and axon terminal sides [17,18,105]. Compared to APOE3-expressing microglia, the APOE4-expressing microglia have a reduced feedback response to neuronal activity due to impaired lipid homeostasis, which promotes the release of pro-inflammatory signals from APOE4-expressing microglia [106].

Therefore, in AD, the normal functions of microglia immune cells (the removal of apoptotic or necrotic neurons, the pruning of non-functional synapses, the production of molecules that support neuronal survival and the prevention of excessive neuronal activation (e.g., epilepsy)) are progressively shifted toward the destruction of functional synapses and neurons [107,108].

## 3. Transmission of Brain Action Potentials in Alzheimer’s Disease

### 3.1. Synaptic Transmission and Plasticity

Hebb’s axiom of synaptic activity and plasticity (i.e., cells that fire together, wire together), complemented by further work summarised with the phrase “cells out of sync lose their link”, has been repeatedly validated and can be applied to brain changes in AD wherein the progressive weakening and elimination of individual synapses adds-up at the whole brain level to altered neural oscillations (i.e., brainwaves) and a progressive imbalance between excitatory (increasing the probability of a postsynaptic action potential) and inhibitory (decreasing the probability of a postsynaptic action potential) postsynaptic potentials in brain networks. Optimal plasticity/activity of brain excitatory synapses (mostly glutamatergic) is essential for memory, learning and other cognitive brain activities [109,110].

Fast excitatory neurotransmission in the brain is mediated by the postsynaptic NMDA (essential for synapse development and plasticity) and AMPA receptors primarily studied in animal models of hippocampal pyramidal neurons. The number of AMPA receptors on the postsynaptic membrane is regulated, in an activity-dependent manner, by NMDA receptors following the depolarisation of the presynaptic membrane and glutamate release. An increased number of AMPA receptors (sourced from recycling endosomes and increased AMPAR synthesis elicited by activation of CaMKII) is associated with LTP. A decreased number of AMPA receptors (due to dynamin-dependent endocytosis of AMPARs from the postsynaptic membrane) is associated with LTD. Postsynaptic LTD (reduction in postsynaptic electrical activity elicited by repeated, low-frequency stimuli) and postsynaptic LTP (increase in electrical postsynaptic activity elicited by repeated, high-frequency stimuli) are examples of synaptic plasticity in hippocampal pyramidal neurons [111,112,113,114]. LTP and LTD are reflected in measurable dendritic shape, size, and number changes. However, the relationship between LTP/LTD on the one hand and all of the observed changes in synaptic morphology on the other are not exactly proportional [109,115].

### 3.2. Synaptic Transmission and Plasticity during Ageing

In humans, a small but measurable decline in memory and learning occurred with normal ageing and was attributed to changes in synaptic plasticity in the atrophied hippocampus and entorhinal cortex. These observations were confirmed in animal brain ageing models where an increased threshold for LTP and a decreased threshold for LTD were recorded and associated with perturbed Ca^2+^ homeostasis. The fundamental electrical properties of nerve membranes, e.g., resting membrane potential, membrane resistance, and width and amplitude of the action potential, do not change over the lifespan [18]. Animal and human studies reported that cell death in the hippocampus and neocortex was not a characteristic feature of normal brain ageing. Normal brain ageing in mammals was consistently associated with spatially discrete changes in synapse morphology (e.g., decreased dendritic branching), reduced dendritic spine density and a reduced number of synapses. Brain ageing increases the probability of AD and overt cognitive decline, since age-related imbalances in synaptic plasticity and transmission are amplified by the development of Aβ and tau pathologies [18].

### 3.3. Synaptic Plasticity during Alzheimer’s Disease

Decreased synaptic density in the hippocampus (due to loss of afferent entorhinal cortex connections), combined with cognitive decline, is the hallmark of AD. The clinical progression of MCI to AD dementia is accompanied by a further reduction in the number of dendritic spines and a reduction in the size and complexity of the dendritic tree of hippocampal region CA1 (CA1) neurons [116]. In AD animal models, a compensatory increase in the size of residual synapses was reported to accompany the reduced number of synapses [117]. On the one hand, animal studies of AD reported a spatial association between Aβ plaques and NFT and reduced dendritic density, length, and complexity [116,117,118,119,120,121,122,123]. AD dysregulation of Aβ metabolism progressively attenuates the expression of proteins regulating (a) presynaptic (e.g., vesicle trafficking by synaptophysin and synaptogyrin, vesicle metabolism by synaptotagmin and syntaxin) and (b) postsynaptic activity (e.g., excitation of the postsynaptic membrane by PSD-95 and drebin) [116]. The decreasing levels of synaptic proteins were associated with concomitant clinical signs of progressive cognitive impairment, from modest to severe cognitive impairment [124,125].

#### LTP and LTD in Alzheimer’s Disease

In animal models of AD, the attenuation of LTP at excitatory synapses precedes Aβ deposits. High levels of AβOs attenuate LTP, decreasing dendritic spine growth and synaptic density. These synaptic changes inhibit memory formation and learning and are the first step in developing memory and learning cognitive impairments in AD. AβOs promote LTD at excitatory synapses. Paradoxically, low levels of AβOs stimulate LTP. AβOs change the probability of postsynaptic LTP or LTD response by modulating Ca^2+^ current through two LTP or LTD-promoting subtypes of NMDARs (LTP is facilitated by a high, and LTD by a low Ca^2+^ current), and by modulating several signalling pathways and postsynaptic receptors. AβOs activate caspases and calcineurin, attenuate cAMP response element-binding protein (CREB) expression and bind to nicotinic acetylcholine receptors. AβOs activation of calcineurin or binding with nicotinic acetylcholine receptors attenuates NMDA receptor expression on the postsynaptic membrane, and this reduced receptor expression promotes AMPA receptor internalisation and LTD. Soluble Aβ products activate caspase-3, and activated caspase-3 dephosphorylates AMPARs, promoting AMPARs’ internalisation. Attenuated CREB expression with soluble Aβ products reduces the expression of proteins necessary for LTP [70,126,127].

### 3.4. Large-Scale Brain Networks’ Changes in Alzheimer’s Disease

#### 3.4.1. Introduction

Hyperexcitability of brain neurons in the early, prodromal stages of AD was reported in laboratory models of AD disease and in patients with LOAD and familial Alzheimer’s disease (FAD) [128,129]. For example, studies of AD animal models reported hyperactive hippocampal neurons that predated a substantial amyloid-β plaque load [93,130]. Direct support for neuronal hyperactivity in patients in the early stages of AD was provided by EEG and magnetoencephalography (MEG) studies that also reported an increased risk of epilepsy in patients developing AD-associated dementia [131,132,133,134]. Additionally, results of fMRI human brain studies are consistent with early stage neuronal hyperactivity in the hippocampus, and large-scale brain networks of AD patients with MCI [93,130,135,136,137]. Several factors contribute to the observed nerve activity, including dyshomeostasis of factors such as intracellular Ca^2+^, N-methyl-D-aspartate receptors (NMDAR), amyloid-β and tau metabolism, glial cells, and inhibitory interneurons, and genetic risk factors for LOAD [128]. The hypothesis that neuronal hyperexcitability could be reflected in altered global brain electrical activity (measured with EEG and EMG) was tested in a whole-brain computational network model that followed human structural brain topology and included brain activity data from patients with AD. The results of computer model simulations support the hypothesis that early stage neuronal hyperactivity underpins the global brain excitation–inhibition imbalance and the large-scale network dysfunction detected on the EEG and EMG records of patients in the early stages of AD [138].

The effects of toxic AβOs and other genetic and non-genetic risk factors on synapses in discrete brain regions (e.g., hippocampus) are also reflected at the whole brain level, where neurons from different brain regions contribute to the activity of large-scale brain networks. Normal activity of large-scale brain networks (also known as intrinsic networks) underpins human cognitive functions such as selective attention, planning, cognitive flexibility and working memory [139]. For example, normal working memory (essential for selective attention, reasoning, decision making and behaviour) depends on functional brain networks that connect discrete brain regions, i.e., the prefrontal cortex on the one hand, with the hippocampus, the perirhinal and parahippocampal cortices, and the posterior parietal cortex on the other [140].

Large-scale brain networks (i.e., intrinsic brain networks) connect brain regions based on functional connectivity elucidated by statistical analysis of fMRI, EEG, PET or MEG brain signals performed during a specific cognitive task (e.g., evaluation of selective attention). Therefore, these brain networks are highly task-dependent and depend on the recording modality (fMRI or EEG), recording parameters, and statistical method (e.g., cluster, independent spatial component) for brain signal analysis. The first attempt to define the core functional large-scale brain networks was published by Mesulam [141]. Large-scale neurocognitive networks distribute processing of attention, language, and memory [142]. Recently, to further facilitate standardisation, comparison, data sharing and integration of large-scale brain networks’ experimental data, six networks were identified as core large-scale brain networks:

- the default mode (DMN) or medial-frontoparietal (one of many resting-state networks associated with self-directed cognitive processes such as introspection and autobiographic memory and characterised by low-frequency, spatially coherent brain activity at a low temporal frequency) [143,144];
- the salience (SN) or mid-cingulo-insular (which directs attention by identifying the vital event, thus preventing goal-driven engagement from being distracted by non-relevant stimuli; it is active during attention, motivation, and executive function tasks. The SN acts as a “switch” that prevents simultaneous activation of DMN and control networks, i.e., it activates the control network and simultaneously deactivates the DMN to support efficient cognition) [145];
- the attention, i.e., dorsal-frontoparietal network (which controls the conscious focus, i.e., attention) [146];
- the control, also known as the central executive or lateral-frontoparietal network (which initiates and modulates cognitive control) [147];
- the sensorimotor or pericentral network (which processes somatosensory information and coordinates motion) [148]; and
- the visual or occipital network (which processes visual information) [149].

In humans, there is significant inter-individual variability in the size, location, and spatial arrangement of the six proposed large-scale brain networks [150,151].

#### 3.4.2. Examples of Changes in Large-Scale Networks in Ageing and Alzheimer’s Disease

Brain ageing is the relative decline in cognitive skills capacity compared to the maximal level experienced in young adulthood. This decline in mental ability is below the level of MCI and does not limit an individual’s ability to lead an independent life. Some individuals (60–80 years old), i.e., “super agers”, have a memory ability equal to or better than individuals in the 20–30 age group when tested on memory recall tasks. Two recent studies compared large-scale brain networks (the default mode network and the salience network), their associated anatomical brain regions, and scores on visual-verbal recognition memory tasks between young adults and aged individuals with conserved memory ability. The conclusions of the studies were:

- some cerebral cortical regions and the hippocampal volumes were indistinguishable between the two age groups, and both groups had thicker cortical areas than the brains of average older adults;
- the hippocampal volume and the thickness of the anterior temporal cortex, rostral medial prefrontal cortex, and anterior midcingulate cortex correlated with memory performance on memory recall tasks;
- aged adults with conserved memory ability had more robust connectivity with the default mode and the salience networks compared to typical older adults, and similar connectivity compared with young adults; and
- the strength of internal connectivity, measured with fMRI in the two large-scale brain networks, correlated with the individual’s performance on memory recall and visual–verbal recognition memory tasks [152,153].

Structural and functional brain connectivity markers were evaluated as potential markers to predict memory performance in normal older adults. Structural brain connectivity was assessed with MR diffusion tensor imaging (DTI) to measure radial diffusivity. Functional brain connectivity was assessed with R-fMRI to evaluate the resting state of five large-scale brain networks. The results of structural and functional brain evaluations were correlated with the adults’ delayed episodic memory scores, their cerebrospinal fluid (CSF) markers of tau and amyloid pathology, and their entorhinal cortex and hippocampus grey matter volumes. The study concluded that fornix and hippocampal cingulum RD and salience network functional connectivity were each independent predictors of memory performance, in contrast to CSF markers and grey matter volumes, which were not [154].

The parietal memory network (PMN), a posterior part of the DMN, was characterised by resting-state fMRI and task activations as a functionally distinct brain network, as recognition memory activates the PMN and autobiographical memory activates the DMN. The DMN and PMN in 36 patients with clinical signs of AD were evaluated with resting-state fMRI and an independent component analysis algorithm. Compared to 43 age-, sex- and education-matched healthy controls, patients with AD had reduced intrinsic functional connectivity, consistent with their neuropsychological assessment score and comparable to changes in their DMN’s functional connectivity [155].

A longitudinal, 9-year study on 265 older adults (baseline age 45–86 years) evaluated, with structural and functional MR imaging, the contribution of educational attainment to the risk of dementia. Cognitive and functional behaviours were assessed by scoring memory, orientation, judgment, problem-solving, community affairs, home and hobbies, and personal care. From the scores of these categories, a clinical measure of dementia severity was derived. Adults aged 65 years without a college degree had a significantly higher large-scale functional brain network resting-state system segregation (loss of large-scale active brain connections) compared to same-age college-educated peers. The observed decline in large-scale active brain connections predicted the severity of the future cognitive decline. The prognostic value of declining large-scale functional brain connection was not correlated with APOE-related genetic risk for AD, levels of cortical amyloid and CSF phosphorylated tau or cortical atrophy [156].

A recent multimodal study compared functional (with resting-state fMRI) and structural (with diffusion MRI) connectivity networks between 46 patients with AD and 39 matched healthy controls. The simultaneous comparison of spatial organisation between functional and structural connectivity networks was performed with graph-theory analysis. Compared to healthy controls, patients with AD had disrupted functional and structural connectivity networks. In patients with AD, the correlation between the active and structural connectivity networks was increased in the default mode network [157].

The standard functional connectivity within and between the three large-scale brain networks was assessed. The default mode, central executive and salience networks are dysregulated in AD and are reflected in cognitive and behavioural abnormalities of patients with AD. Regular brain activity, segregated into large-scale networks with internal and external functional connectivity, is essential for maintaining the energy efficiency of the brain’s metabolism. The effect of dysregulated functional connectivity of these large-scale brain networks on brain metabolism was evaluated with fluorine-18 fluorodeoxyglucose PET imaging (FDG-PET) and fMRI study, and compared to normal controls and patients with MCI. The researchers concluded that the impaired segregation of the salience network functional connectivity, uncoupled with glucose metabolism, contributes to cognitive decline in patients with AD [158].

Patients with AD or the behavioural variant of frontotemporal dementia (bvFTD) have distinct changes in large-scale brain networks. Compared to normal controls, patients with AD had a lower integration in the default and control networks, and patients with bvFTD had a lower integration in the salience network. The scale of observed changes in the intrinsic networks of patients with AD or bvFTD was associated with the severity of attention deficits and neuropsychiatric symptoms [159].

## 4. Quantifying Synaptic Function and Density

### 4.1. Introduction

In recognition of recent advances in brain imaging techniques and AD aetiology, the National Institute on Aging and the Alzheimer’s Association published a research framework for AD. This framework shifts the basis of AD diagnosis in living patients from clinical signs and symptoms to ATN biomarkers (β-amyloid (A), phosphorylated tau (T) and neurodegeneration (N) markers), measured with functional brain imaging techniques (MRI, FDG-PET, PET imaging of synaptic vesicle glycoprotein 2A (SV2A) radioligands, single-photon emission computerised tomography (SPECT) and quantitative EEG) and in blood or CSF samples. The proposed framework does not claim a causal link between ATN biomarkers and AD pathogenesis. The framework recognises the temporal association of these biomarkers with the development and progression of cognitive decline in patients with AD. Therefore, ATN biomarkers can, with sufficient sensitivity (range 73–89%)) and accuracy (range 70–85%), identify subjects with a high risk of transitioning from MCI to AD dementia, and also promote the comparison, sharing, and aggregation of AD biomarkers’ research data. Some research suggests that CSF biomarkers (e.g., the Aβ42/Aβ40 ratio) have a higher sensitivity for detecting presymptomatic AD than either SPECT, FDG-PET or MRI. Large-scale, multicentre studies with harmonised study protocols will be necessary to give a definitive answer [61,160,161].

#### Synaptic Density and Synaptic Function are Related and Not Equivalent Terms

In vivo experiments in mammals illustrate the differences between synaptic density and function. For example, rat brain images with PET radiotracers (4R)-1-((3-Fluoranyl-4-pyridyl)methyl)-4-(3,4,5-trifluorophenyl)yrrolidine-2-one ([18F]UCB-H), a synaptic density marker (measuring uptake at SVA2 synaptic protein) and an [18F]fluorodeoxyglucose ([18F]FDG) synaptic function marker (measuring glucose metabolism) acquired 24 h apart on the same animal do not overlap in all brain areas. The differences were most pronounced in the prefronal cortex and cerebellum, where the glucose metabolism rate was relatively higher compared to the radiotracer-measured SV2A synaptic density concentration. On balance, synaptic density markers are less susceptible to changes induced by the experimental protocol (e.g., to anaesthetics for PET and MRI scanning) than synaptic function markers. On the other hand, synaptic function markers are more sensitive to changes in the overall and regional differences in brain electrical activity, glucose concentration and the level of blood oxygenation [11].

### 4.2. Synaptic Density Quantification with High-Resolution Electron Microscopy

Despite the recent advances in brain imaging of living patients with AD, the gold standards for evaluating the quality and quantity of brain synapses in samples of post-mortem tissues are high-resolution electron microscopy (EM) (transmission electron microscopy (TEM) or scanning electron microscopy (SEM)) and immunohistochemistry (IHC). Both EM or IHC do not allow for a longitudinal assessment of AD-related brain pathology or response to treatment interventions. However, due to their high spatial resolution, these methods enable a direct and specific evaluation of the synaptic structure and spatial organisation, the density and morphology of dendritic spines, and the expression of synaptic proteins in the pre-synaptic and post-synaptic membranes. For example, the number of docked presynaptic vesicles is visualised with TEM, and a 3D image of dendritic spines is constructed from SEM scans. EM studies of AD animal models investigated synaptic structures and their regional densities. They confirmed a decreased synaptic density in the hippocampal dentate gyrus or the entorhinal cortex brain regions, known to be affected by AD pathologies. The trade-off for a high resolution in EM is a reduced field of view and a small sample size [11]. IHC and IF have advantages over EM, such as broader accessibility and lower cost; they are used to evaluate (a) the structure and density of dendritic, post-synaptic spines in predominantly excitatory synapses (due to their importance in synaptic plasticity, learning and memory processes) and (b) to map the expression of presynaptic and postsynaptic proteins with specific antibodies [11,162,163]. Since dendritic spines are present in most synapses, the number of visually detected spines (per micrometre of dendrite) represents an index of synaptic density and activity. Minor changes in spine morphology can modulate synaptic transmission, increase synaptic strength, and increase the number and size of dendritic spines [164,165,166,167,168]. IHC enables the differentiation of excitatory and inhibitory synapses, with antibodies selectively targeting either proteins predominantly expressed in excitatory synapses (e.g., PSD-95) or in inhibitory synapses (e.g., gephyrin) [169,170,171,172]. Most synaptic proteins (e.g., SVA2) are expressed in excitatory and inhibitory synapses, enabling a global brain mapping and quantifying synaptic density [171,173,174].

### 4.3. Synaptic Density Quantification with Histology and Immunohistochemistry

The study of synapses in animal models with histological (HT) and immunohistochemical techniques (IHT) associated the genetic, environmental, and life-span changes in brain synaptic protein expression, morphology, density, and plasticity with cognitive changes [175,176,177,178]. In AD models, Aβ protein brain pathology was associated with decreased spine density and dendritic spine morphological changes. Increased calcineurin activity, attenuating the peptidyl-prolyl isomerase Pin1 signalling pathway, was associated with Aβ plaque brain pathology [179,180,181]. Decreased synaptic spine density, accompanied by cognitive impairment, precedes brain Aβ plaque accumulation [182]. HT and IHT quantify brain synapses with dendritic spines only; these spines are absent in most inhibitory neurons. Additionally, the IHT pre-/postsynaptic protein quantification established on chemical synapses does not include (a) synaptic proteins in electric synapses that have an essential function in local inhibitory circuits of the adult brain [11,32,183], or (b) the presynaptic proteins in astrocytes and microglia that seem to regulate the exocytosis, for example, of glutamate-containing vesicles [11,184,185]. The introduction of 3D stimulated emission depletion microscopy and super-resolution shadow imaging has improved the visualisation and quantification of dendritic spine structure and density in 3D [186,187,188].

### 4.4. Synaptic Density Quantification with Pre- or Post-Synaptic Proteins in Blood or CSF

#### 4.4.1. Established Synaptic Protein Markers Measured in Blood or CSF

Biomarkers for AD-related synaptic dysfunction have been divided into pre- and postsynaptic groups depending on the protein’s synaptic localisation. Recent advances in mass spectrometry and immunoassay techniques have enabled reliable synaptic protein quantification of CSF and blood samples in living patients. The most assessed protein biomarkers for synaptic density quantification are (a) pre-synaptic growth-associated protein 43 (GAP-43), (SNAP-25), and synaptotagmin-1, and (b) postsynaptic protein neurogranin [17,20,66].

GAP-43, located on the cytoplasmic side of the plasma membrane, is abundantly expressed in the adult brain’s hippocampus, entorhinal cortex, and neocortex, contributing to normal memory formation and storage. Phosphorylated GAP-43 interacts with synaptophysin and SNAP-25 to facilitate synaptic vesicle recycling. Compared to controls, increased GAP-43 CSF levels were detected in patients with AD and reduced CSF levels in patients with PD; blood levels were reduced in patients with AD and MS. SNAP-25 protein is essential for normal vesicular exocytosis, neurite outgrowth, and LTP. Increased CSF levels were detected in patients with AD (already at the very early stage), PD or sporadic Creutzfeldt–Jakob disease (CJD). Synaptotagmin-1, a Ca^2+^-sensor transmembrane vesicle protein, contributes to fast and synchronous vesicle fusion (i.e., neurotransmitter release in the synaptic gap) in hippocampal neurons in response to increased intracellular Ca^2+^ concentrations. Compared to controls and patients with AD, the most increased CSF levels were detected in patients with MCI related to AD [17,20,66]. Neurogranin is an intracellular protein concentrated in synaptic spines. The binding of this protein to the Ca^2+^-signalling mediator calmodulin promotes memory formation. Patients with prodromal and overt AD or CJD have increased CSF levels compared to controls [17,20,66].

Increased CSF concentrations of neurogranin fragments predict cognitive decline, brain atrophy, and reduced glucose metabolism for the early stages of AD disease [189,190,191,192]. Neurogranin is the most extensively studied synaptic protein marker for detecting AD. There is an urgent need to develop additional postsynaptic protein markers, since there are no established synaptic protein markers to evaluate the early AD-related decrease in glutamatergic synapses, the prevalent excitatory synapses in the brain [17,193,194,195,196,197].

A cross-sectional study of middle-aged, cognitively unimpaired female and male participants evaluated CSF levels of synaptic protein markers neurogranin, GAP-43, SNAP-25 and synaptotagmin-1 in conjunction with CSF Aβ42/40 ratio, Aβ PET values, and CSF levels of P-tau, T-tau, and NFL. The primary study conclusions were:

- CSF levels of all synaptic protein markers and Aβ pathology increased with age;
- CSF levels of synaptic protein markers were increased even in individuals with low levels of CSF Aβ42/40 and Aβ PET values;
- female participants had higher CSF neurogranin values; APOE4 participants had the highest values for CSF SNAP-25; and
- higher CSF synaptic biomarkers correlate with higher CSF p-tau and NFL values [198].

#### 4.4.2. Emerging Synaptic Protein Markers Measured in Blood or CSF

About 100 emerging synaptic protein markers for AD were identified in the CSF samples of living patients. They include, among others: neuregulin 1, neurofascin, 14-3-3 proteins, synaptic proteins from plasma-derived exosomes (e.g., neurexins, member of the Ras superfamily small G proteins (Rab) family, synaptotagmin-2, glutamate receptor 4, synaptophysin), neurofilaments (the light-chain fragment), neuronal pentraxins and SVA2 [20,199].

Blood-derived exosomal quantities for synaptic biomarkers GAP43, neurogranin, SNAP25 and synaptotagmin 1 correlated with their CSF values and detected patients with preclinical AD 5 to 7 years before they developed cognitive impairment. Compared to controls, patients with AD had reduced levels of these neuronal biomarkers in CSF and blood-derived exosomes [200].

From the blood of patients with AD, six exosomal miRNAs (adjusted for age, sex, education years, and APOE ε4 status) were identified, which were either upregulated (miR-29c-5p, miR-143-3p, miR-335-5p, and miR-485-5p) or downregulated (miR-138-5p and miR-342-3p) compared to controls. These exosomal changes could predict AD 5 to 7 years before the onset of cognitive impairment [201].

The light-chain neurofilament fragment was suggested as a candidate for neuronal death, since its increased blood and CSF levels are in good agreement across many brain disorders [66]. The postsynaptic neural pentraxin-2 regulates synaptic plasticity by binding to AMPAR on the postsynaptic membrane of excitatory synapses. Compared to controls, neural pentraxin-2 levels in CSF samples are reduced in patients with MCI or AD, and this reduction is reflected in cognitive decline and reduced hippocampal volume [20,202]. Recently, an observational, retrospective, multicentre study was implemented to evaluate neural cell adhesion molecule (NCAM)/amphiphysin from dual-labelled exosomal proteins and microRNAs (miRs) isolated from peripheral blood as markers for early diagnosis of AD. Patients with SCI, amnestic MCI (aMCI), AD dementia, and vascular dementia (VaD), and 40 healthy controls were evaluated for tau pathology, Aβ pathology, NFL, NCAM/amphiphysin one from dual-labelled exosomal proteins, and microRNAs (miRs) from blood samples. The study concluded that the plasma NCAM/amphiphysin one dual-labelled exosomal miR-29c-3p had the same diagnostic power as the CSF biomarkers and could assist in diagnosing SCI [203].

Combining shotgun proteomics of CSF samples with (a) analysis of the literature and a database search and (b) another selection with selected reaction monitoring yielded a list of nine synaptic proteins evaluated for specific expression at the human brain synapse. Evaluation of these proteins in CSF samples of patients from three independent clinical cohorts yielded six synaptic biomarkers, candidates for novel CSF biomarkers of synapse loss, that changed in preclinical AD patients before markers of neurodegeneration [204].

SV2A is a synaptic vesicle transmembrane protein located in dense-core and small synaptic vesicles. It is assumed to participate in regulating neurotransmitter release, synaptotagmin transport and several extra synaptic functions (details in 4.1.). The protein has been suggested as a global marker for synaptic density, since it is widely expressed in normal brain neurons. It was reduced in brain disorders, with a decline in synaptic density [17,205,206,207,208,209]. SV2A-based evaluation of synaptic density is performed with PET imaging of the protein’s radioligands. The use of SV2A content in samples of CSF as a marker for synaptic density, and the correlation with its radioligands for PET imaging, is currently under development [20].

### 4.5. Synaptic Density Quantification with Aβ4 and Tau in the Blood or CSF

Rising Aβ4 and tau levels in the blood or CSF are indirect measures of a progressively reduced brain synaptic density. These values start increasing in cognitively normal patients with AD and rise over a decade in parallel with the development of MCI and later to overt AD with dementia [66].

In AD patients, Aβ42 content in CSF was reported to be either reduced or increased before the onset of dementia [66]. The CSF Aβ42/P-tau or Aβ42/Aβ40 ratios with the addition of total tau and phosphorylated tau measurements have been proven more reliable markers for the presence and progression of cognitive decline from MCI to overt AD than CSF Aβ42 alone [210,211,212]. The plasma amyloid-β precursor protein isoforms (APP) 669–711/Aβ42 and Aβ40/Aβ42 ratios improved the prediction of brain amyloid in patients with AD [213]. Additionally, plasma-derived neuronal vesicles enriched with Aβ42 could reliably predict cognitive decline in patients with AD [214].

Compared to healthy controls, patients with AD had significantly increased T-tau and P-tau levels in samples of CSF [215,216,217,218]. P-tau levels were significantly increased, even in the preclinical AD stage when patients had a concurrent, minimal presence of Aβ pathology [219]. Increased CSF tau and plasma-derived neuronal vesicles enriched with P-tau and total-tau can predict cognitive decline in patients with AD [214,220,221]. P-tau levels in plasma can reliably differentiate between AD and non-AD dementias [222]. The sodium-dependent phosphate transport protein 2A (NPT2)/phosphorylated tau protein (P-tau) ratio in CSF strongly correlated with the level of cognition and predicted cognitive decline in patients during the transition from MCI to overt AD [223].

There is a significant overlap of plasma T-tau levels between healthy cognitive controls, individuals with MCI and patients with AD. However, plasma p-tau181 correlated with tau PET in Aβ-positive AD individuals, and could accurately distinguish between healthy controls and MCI individuals with a positive Aβ-PET scan [222,224,225].

### 4.6. Synaptic Density Quantification with PET

#### 4.6.1. Introduction

Synaptic density measurement in the living human brain with PET was made possible with in vitro and ex vivo brain autoradiography which used radioisotope-labelled molecules in mammalian animal models to characterise the metabolism, ligand selectivity and target localisation of radiotracers. The development of presynaptic SV2A radiotracers was the key to synaptic density quantification, first with autoradiography and later with PET, and to evaluation of the effect of different treatments [226,227,228,229,230,231,232,233]. The lower spatial resolution of PET imaging compared to autoradiography (mm for the former and um for the latter) is compensated for by the PET’s advantage in quantifying the number of synapses over time in the same living patient, which enabled the development of biomarkers for neurodegenerative brain disorders [11].

#### 4.6.2. SV2A PET Tracers

PET brain imaging of the pre-synaptic, transmembrane vesicle glycoprotein 2 (SV2) with radioligands derived from the anti-epileptic drug levetiracetam (with a specific, but relatively low binding affinity for SV2) enables visualisation of synapses and quantification of synaptic density in vivo. For example, compared to age-matched controls, patients with AD had an attenuated PET SV2 radioligand signal [205]. SV2 is ubiquitous in axon terminals of inhibitory and excitatory CNS neurons (i.e., GABAergic and glutamatergic neurons), regulates neurotransmitter release and is concentrated at the whole brain level in grey matter regions, where neural soma and synapses between the axon terminals and dendrites are located. SV2-A is the predominant isoform, with the highest concentration in the basal ganglia and thalamus, and SV2-B is preferentially expressed in the cortex and hippocampus. Pyramidal and hippocampal neurons express SV2-A and SV2-B isoforms. The expression of the SV2-C isoform is absent in the neocortex, hippocampus, and thalamus; its location is mapped to the striatum, substantia nigra, pons, medulla oblongata, and olfactory bulb. The expression pattern of SV2 isoforms has neither been associated with the transport of specific neurotransmitters nor with the expression patterns of other synaptic proteins, suggesting similar brain functions [17,234].

SV2 proteins modulate (a) synaptotagmin content in synaptic vesicles with Ca^2+^-dependent or Ca^2+^-independent interactions and (b) the release of neurotransmitters. SV2 proteins regulate the presence and quantity of synaptotagmin in synaptic vesicles by modulating synaptotagmin’s transport to the vesicles and synaptotagmin’s uptake during endocytosis. SV2 proteins promote neurotransmitter release by priming the vesicles for Ca^2+^-induced exocytosis. To fully understand the direct and indirect roles of SV2 proteins in synaptic transmission and assure a correct interpretation of the PET SV2 radioligand signal in the brain, it is essential to note that this protein participates in diverse cellular processes, including MT membrane fusion, galactose transport, and interactions with ATP and extracellular proteins. Therefore, combined measurements of ATN biomarkers in a single patient with AD further increase the probability of identifying subjects with a high risk of transition from MCI to AD dementia [17,234,235].

New SV2A PET tracers are being continuously developed for in vivo quantification of SV2A brain levels in animal models and patients with AD to optimise the tracer’s (a) binding affinity for SV2A, (b) brain uptake, (c) metabolism, (d) in vivo kinetics, and (e) half-life, which should neither require on-site cyclotron tracer synthesis nor be too long [11,234,236,237,238,239,240,241].

#### 4.6.3. Evaluations of PET Radiotracers for Brain Synaptic Density Quantification in Mammals

Hippocampal synaptic loss in animal models of AD and the effect of potential treatment interventions were only recently quantified with SVA2 PET tracers [241,242,243]. These studies have identified several key issues relevant to animal and human brain imaging with SV2A radiotracers:

- selection of a protocol-appropriate radiotracer injection method (intravenous or intramuscular);
- quantifying radiotracer binding either from multiple blood samples at precise time points or by non-invasive methods, e.g., the use of a reference region with no specific uptake;
- minimising the mass effect on PET imaging during multiple sequential scans, which is especially relevant for small animal studies;
- the use of reconstruction algorithms to reduce image resolution (e.g., by smoothing);
- the need to address the variable disease-related changes in the pre- and postsynaptic protein content of different brain regions;
- SVA2 expression’s correlation with the number of synaptic vesicles in the presynapse; this number is also dependent on synaptic (brain) activity, not only on synaptic density; and
- radiotracers’ binding affinity for SV2A, which could change across the presynaptic exocytosis process due to modifications of SV2A’s conformation and electrostatic properties [244,245,246].

SVA2 PET tracers can quantify synaptic density in longitudinal studies that evaluate the effects of therapeutic interventions developed to attenuate brain synaptic loss due to toxic AβOs and phosphorylated tau products [234,243,247,248,249].

#### 4.6.4. Evaluations of PET Radiotracers for Brain Synaptic Density Quantification in Non-Human Primates

In 2016, the SV2A PET radiotracer (4R)-1-[(3-(11C)Methylpyridin-4-yl)methyl]-4-(3,4,5-trifluorophenyl)pyrrolidin-2-one ([11C]-UCB-J)’s synthesis and characterisation for synaptic brain imaging in nonhuman primates were reported. The radiotracer had high affinity and selectivity for SV2A, enabling excellent brain imaging and optimal pharmacodynamic characteristics; its drawback was a limited usage time window due to a short radioactive half-life of about 20 min [250]. This SV2A PET radiotracer is the gold standard for brain synaptic density quantification [234]. First-in-human evaluation of the PET radiotracer [11C]-UCB-J is discussed in Section 4.6.5. Fluorination of [11C]-UCB-J produced an SV2A PET radiotracer [18F]-UCB-J with a longer radioactive half-life (110 min), and thus broader application in synaptic density evaluation of nonhuman primates that also retained the optimal brain imaging and pharmacokinetic (i.e., absorption, distribution, metabolism, and excretion) characteristics of its precursor [11C]-UCB-J [237]. The extended radioactive half-life of UCB-J F-18 derivatives enables multicentre clinical trials between centres with on-site cyclotron PET radiotracer production and brain imaging centres that lack PET isotope production facilities.

Recently, an 18F-labeled difluoro-analogue of UCB-J (i.e., 18F-SynVesT-1 known as 18F-SDM-8) was developed and compared to [11C]UCB-J for dosimetry calculations in nonhuman primates and test–retest reliability in healthy human subjects. The authors concluded that 18F-SDM-8 is appropriate for brain synaptic density quantification in humans due to its reproducible fast and high brain uptake, appropriate pharmacodynamics, and high levels of specific binding [251]. A first-in-human study followed this study to evaluate the kinetic and binding properties of (4R)-4-(3-(18F)fluoranyl-5-fluorophenyl)-1-[(3-methylpyridin-4-yl)methyl]pyrrolidin-2-one (18F-SDM-8) and compare them with [11C]-UCB-J. The study concluded that in the living human brain, 18F-SDM-8 has fast and reversible kinetics and high specific binding to SV2A, both essential for imaging and quantifying synaptic density in neuropsychiatric disorders [252].

Constantinescu et al. [238] developed and evaluated several [18F] derivatives of UCB-J and UCB-H for brain synaptic density quantification in non-human primates. They identified the UCB-J derived 18F analogue of [11C]UCB-J ([18F]MNI-1126) tracer as the best fluorinated SV2A PET radiotracer that combined a long half-life with imaging characteristics comparable to [11C]UCB-J. To further optimise fluorinated SV2A PET radiotracers for synaptic brain imaging, Constantinescu et al. [218] developed a UCB-A-based PET radiotracer (4R)-4-(3-(18F)fluoranyl-5-fluorophenyl)-1-[(3-methylpyridin-4-yl)methyl]pyrrolidin-2-one ([18F]SDM-16) and compared it for brain imaging quality and pharmacodynamics with [ 11C]UCB-A, [11C]UCB-J, [18F]UCB-H, and [18F]SynVesT-1 PET radiotracers. Compared to these PET radiotracers, the [18F]SDM-16 PET radiotracer had the highest and reversible SV2A specific binding, a relatively low nonspecific binding in white matter, and the most increased metabolic stability in non-human primate brains [253].

#### 4.6.5. Evaluations of PET Radiotracers for Brain Synaptic Density Quantification in Humans

The first published study to compare hippocampal synaptic density between age and sex-matched cognitively normal participants and patients with AD was performed with [11C]UCB-J PET radiotracer imaging. In this pilot, cross-sectional study, participants were ten patients with AD (all with β-amyloid-positive PET scans and variable cognitive impairment from MCI to mild dementia) and 11 cognitively normal participants (all with β-amyloid-negative PET brain scans). Compared to cognitively normal participants, patients with AD had a significant brain atrophy-corrected reduction in hippocampal SV2A-specific binding. There was a statistically significant correlation between lower hippocampal synaptic density values and lower cognitive scores for episodic memory and global functioning from combined measurements of both groups of participants. The authors concluded that large-scale and longitudinal studies are necessary to define the association between cognitive decline and regional brain changes in SV2A-specific PET radiotracer binding [205].

Human biodistribution and dosimetry estimates were calculated with OLINDA software from the data of three healthy subjects injected with the [11C]UCB-J PET radiotracer and subjected to sequential whole-body PET scans for two hours. The study concluded that a single intravenous administered dose of [11C]UCB-J enables multiple PET examinations to be carried out on the same subject without exceeding the annual dose limitations [254].

The brain region-specific reduction of [18F]-UCB-H in humans with AD was evaluated in 24 patients (either with MCI or with positive [18F]-Flutemetamol amyloid PET scans) and 19 healthy controls. MCI or AD patients had a reduced uptake in the basal forebrain, anterior/dorsomedial thalamus and hippocampus compared to controls. The synaptic loss was most pronounced in the hippocampus. According to autopsy-based values for synaptic density in MCI and AD, most of the patients included in the study were in the early stages of AD. Patients with reduced [18F]-UCB-H hippocampal uptake, a measure of synaptic loss, had an associated cognitive impairment and unawareness of memory decline. This unawareness of memory decline could be due to the degradation of normal connectivity in the hippocampus and connecting regions, essential for episodic memory, the memory of everyday events associated with past personal experiences at particular times and places. The observed reduced synaptic density in discrete brain regions suggests that the medial temporal-frontal lobe circuits are affected early in AD [255].

#### 4.6.6. PET Synaptic Density Quantification Studies in the Human Brain with Early Alzheimer’s Disease

[11C]UCB-J PET brain scans in 34 patients with early stage AD detected significant, volume loss-adjusted reductions in SV2A binding in medial temporal and neocortical regions, compared to brain scans of 19 cognitively normal controls. The study concluded that widespread synaptic loss in the human brain is a hallmark of early AD [256].

O’Dell et al. evaluated, in a cross-sectional study with [11C]UCB-J and [11]C-labelled Pittsburgh Compound-B ([11C]PiB) PET radiotracers, the spatial association between Aβ deposition and SV2A synaptic density in the living human brains of patients with AD-associated aMCI or with AD mild dementia. They reported a statistically significant inverse association between global Aβ deposition and hippocampal PET SV2A radiotracer binding in aMCI patients, and no significant association for patients with mild AD dementia. The study results were consistent with the hypothesis that fibrillar Aβ accumulation reaches a plateau in the early clinical stages of AD (i.e., AD with mild dementia), after which other pathological changes may be responsible for propagating the continued synaptic loss [257].

A longitudinal, 2-year follow-up study (two PET brain scans per participant, one at baseline and one after two years), with an 18F-MK-6240 radiotracer for tau quantification and an 11C-UCB-J SVA2 radiotracer for synaptic density evaluation, was implemented in 12 aMCI patients (11/12 were amyloid PET positive). Baseline scans in aMCI patients detected neocortical tau accumulation, which progressed to Braak stage V/VI after two years. Baseline synaptic loss was limited to limbic regions (Braak II/III) and progressed over time, following the tau-specific progression pattern up to the Braak stage IV/V after two years. The observed synaptic density reduction correlated with the patients’ cognitive decline. The authors concluded that in vivo synaptic brain loss follows the pattern of tau accumulation in the brain, and that the observed time delay between the propagation of synaptic loss and tau brain accumulation opens a time window for the use of tau-targeting therapies to attenuate brain synaptic loss [258].

### 4.7. Synaptic Function Quantification

#### 4.7.1. Quantitative EEG Markers

EEG records capture the summed excitatory and inhibitory activity of post-synaptic potentials located mainly in cortical pyramidal neuronal dendrites between cortical layers II, III and V on the one hand and layer one on the other [259,260,261,262]. Therefore, EEG brain activity reflects mainly the post-synaptic potentials from the superficial cortical layers and, to a lesser degree, from deeper brain structures such as the cingulate gyrus or hippocampus. The amplitude of EEG signals depends on thousands of neurons with parallel dendrites with time-synchronous activity in the same direction.

Quantitative EEG markers are used to evaluate cognitive function; for example, the pattern of relative amplitudes of discrete wave frequency intervals, calculated from the total EEG brain activity, reflects the oscillatory activity of the underlying brain network supporting cognition. However, quantitative EEG cannot discriminate among different synaptic dysfunction aetiologies. Despite the method’s drawbacks, quantitative EEG represents the best widely available method for tracking AD disease progression in a clinical setting that correlates with CSF biomarkers for AD [259,263,264,265]. The spectral power of EEG changes in patients with AD towards a progressive amplitude increase in a low, 4–8 Hz frequency range, an early sign of future AD-associated cognitive decline in non-demented amyloid-positive subjects [263,266]. Pharmacological interventions in patients with AD significantly reduced their theta power in the EEG frequency analysis, and improved the patients’ scores on working memory and working memory capacity tests [267].

#### 4.7.2. FDG-PET

Fluorine-18 fluorodeoxyglucose PET imaging enables the visualisation of the brain’s glucose metabolism rates used for ATP-intensive synaptic activity during synthesising, releasing and recycling of neurotransmitter molecules. Thus, FDG-PET can be used as a direct measure of synaptic activity and an indirect measure of synaptic density in longitudinal studies of AD progression, since the longitudinal glucose metabolism decline measured on FDG-PET brain scans precedes and is later associated with the progression of cognitive decline in AD patients [17,268,269,270]. Further large-scale, longitudinal studies are necessary to fully develop FDG-PET imaging for staging AD-associated cognitive decline and evaluating dementia-attenuating therapeutic interventions [17,271].

#### 4.7.3. MR Spectroscopy and GluCEST-MRI

MR spectroscopy (MRS), measuring the metabolite-specific resonant frequency of glutamate-to-creatine ratio to evaluate the synaptic function, is currently in the preclinical development stage. Background research with EM located the highest levels of glutamate in the axon terminals of rat hippocampal excitatory neurons, and a high correlation between glutamate levels and synaptic vesicle density was detected in rat spinocerebellar terminals [11]. However, Onwordi et al. reported that the correlation between the glutamate-to-creatine ratio and the [11C]UCBJ PET radioactive tracer uptake is significant in healthy volunteers’ hippocampus and anterior cingulate cortex, but not in patients with schizophrenia [11,230].

Glutamate MRS sensitivity is limited by the relatively low glutamate brain concentration compared to water molecules, thus generating a weaker signal and lower temporal and spatial resolution. The measurement of low-concentration brain metabolites in vivo is improved by glutamate chemical exchange saturation transfer (GluCEST) MRI, which detects proton chemical exchange between water and some molecules and has an up to two orders of magnitude higher resolution compared to MR spectroscopy (MRS), and which approaches the solution of conventional brain MR imaging techniques [11,272]. Therefore, GluCEST is a promising method for evaluating the synaptic density of glutamatergic synapses but not inhibitory or electric synapses [11]. To enable synaptic density measurements, further improvements in MRS and GluCEST metabolite specificity are necessary, since the signal is generated, in addition to glutamate, by the presence of other metabolites, e.g., creatine and GABA. Additionally, the GluCEST signal amplitude is pH-and magnetic field strength dependent [11,273].

### 4.8. Synaptic Function Quantification with MRI

#### 4.8.1. Structural/Volumetric MRI

Structural MRI brain imaging, combined with image reconstruction algorithms (e.g., a deep learning algorithm), measures cortical thickness, enlargement of the lateral ventricles, and early AD-related brain changes (i.e., regional volume atrophy in the hippocampus and entorhinal cortex) and thus (a) differentiates between brains of healthy controls from brains of patients with AD-related and other dementias or (b) measures and predicts the progression from MCI to AD with high accuracy, sensitivity and specificity [66,274,275,276,277,278,279]. Structural MRI provides an indirect measure of synaptic function and density, since its spatial resolution does not enable the visualisation of synapses [11].

#### 4.8.2. Diffusion Tensor Imaging and Fibre Tractography MRI

Diffusion tensor imaging (DTI) MRI measures the diffusivity of water molecules (which diffuse more freely along the direction of axonal fascicles than perpendicularly to them) to delineate and evaluate the brain white matter with 3D reconstruction technique fibre tractography [278,280]. DTI MRI brain scans predict the conversion from MCI to AD, and distinguish between AD-related and other dementias [278,281,282]. The disadvantage of DTI MRI for clinical use is the technique’s susceptibility to motion artefacts, long scanning time and interpersonal variability [283].

#### 4.8.3. Functional Magnetic Resonance Imaging (fMRI)

Blood oxygenation level-dependent (BOLD) fMRI measures the overall changes in brain blood oxygenation (oxygen consumption firstly decreases the BOLD signal and secondly elicits a compensatory blood flow increase, which leads to a net increase in the BOLD signal) that are assumed to be associated with changes in brain neuronal activity [278]. Three assumptions underpin the interpretation of the BOLD signal:

- in adults, a net increase in neuronal activity is associated with an increased BOLD signal;
- neurotransmitter (e.g., glutamate) release promotes vasodilation of cerebral blood vessels by vasoactive signalling molecules from neurons and glia; and
- the BOLD movement mainly reflects cerebral cortex and cerebellar synaptic activity, which consume more ATP and oxygen than the traffic of action potentials in these brain regions [284].

Patients with MCI and AD have decreased functional connectivity in the DMN during resting-state fMRI brain scans [285]. Compared to normal controls, fMRI brain imaging measured differential changes in functional connectivity between patients with late or early AD. Patients with early AD had increased functional connectivity in the ventral and anterior regions. They had decreased functional connectivity in the posterior DMN, and patients with late AD had reduced functional connectivity in ventral, anterior, and posterior DMN regions [286,287,288].

## 5. Hippocampus, Human Memory Formation and Cognition

### 5.1. Cognition and Memory

The Diagnostic and Statistical Manual of Mental Disorders recognises six major cognitive domains further divided into numerous clinically and experimentally verifiable subdomains. These primary cognitive qualities are:

- complex attention (the ability to focus on multiple things at once, or to select what to pay attention to and what to ignore);
- executive function (which enables planning, prioritising, making decisions, responding to our environment, and moving between tasks);
- learning and memory;
- language (the capability to communicate by writing, reading, or speaking);
- perceptual-motor control (the ability to coordinate the body’s movements in response to environmental stimuli); and
- social cognition (the capacity to control personal desires, express empathy, recognise social cues, read facial expressions, and self-motivate) [289].

The three main memory types in humans, recently revised in [290], are:

- sensory memory (i.e., storing information from the senses);
- short-term memory (a temporary, continuously updated storage for small amounts of incoming sensory information that is further processed by the working memory domains of the central executive, the visuospatial sketchpad, the phonological buffer and the episodic buffer working memory); and
- long-term memory (wherein information is stored for the long term).

Information not transferred from sensory inputs to the long-term memory is lost at the levels of sensory memory or short-term memory processing. Information from long-term memory can be accessed consciously (i.e., explicit memory) or unconsciously (i.e., implicit memory). Explicit (also known as declarative) memory is subdivided into (a) episodic memory (which enables the learning, storage, and retrieval of information on personal and daily time and place-related events, and includes detailed information about the event itself) and (b) semantic memory (composed of factual information on concepts and meanings). The implicit memory domains are procedural memory (motor and executive skills), associative memory, non-associative memory, and priming [290].

Memory assessment experiments have to control or measure viewing behaviour. Viewing behaviour modulates cognitive states (e.g., emotion, attention, curiosity, intentional remembering, and forgetting), thus influencing brain activity and hippocampal memory function measurements. For example, associative processing and viewing behaviour activities can be challenging to distinguish in hippocampal activity associated with memory assessment measurements [291].

### 5.2. Hippocampal Connections Underpinning Explicit Memory

**Figure 5:**
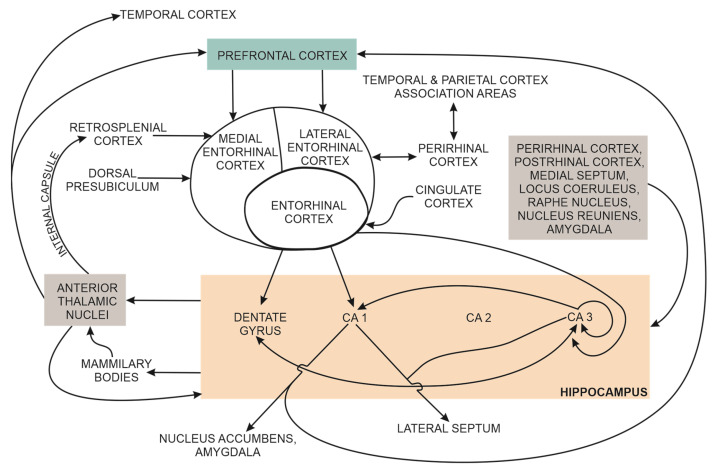
A simplified summary of hippocampal connections in mammals. The dentate gyrus and subfields CA1 to CA3 are functionally and anatomically distinct hippocampal subunits.

Normal hippocampal function is essential for unrestricted semantic and episodic memory. However, compared to episodic memory, the hippocampal contribution to semantic memory needs to be better understood [292]. The critical brain regions that support normal episodic memory function are the perirhinal cortex (active during visual object recognition), the entorhinal cortex, the parahippocampal cortex (active during perception and processing of environment-related information), the neocortical regions near the hippocampus, and the neural circuits within the medial temporal lobe and hippocampus [293]. These regions are connected with two-way projections (Figure 5). Most neural connections between the hippocampus and neocortex go through the entorhinal cortex [290]. The hippocampus is a reference map/coordinate system for storing discrete memories in neocortical regions [294].

Currently, three memory consolidation theories attempt to explain the hippocampal role in memory consolidation based on experimental evidence in animal and human studies. These theories are the standard consolidation theories (SCTs), the multiple trace theory (MTT) and the most recent memory manifold theory (MMT) [295,296].

The SCTs’ postulates are:

- memories are not stored in the hippocampus;
- memories are the product of a time-limited, temporary integrative interaction between hippocampal representations and neocortical information that promotes memory consolidation in the neocortex; and
- after memory consolidation, memory retention and retrieval occur independently of the hippocampus in the neocortex.

SCTs do not explain retrograde amnesia that follows hippocampal damage, do not distinguish between episodic and semantic memory consolidation, and assume that the content of consolidated memories does not change over time [295,296].

The MTT is underpinned by the spatial, long-axis, anterior-posterior hippocampal connectivity specialisation with differential neocortical regions that enable the hippocampus to function as a hub for memory formation and transformation [297,298]. The posterior hippocampal section has preferential connectivity to perceptual and spatial representational systems in the posterior neocortex. The anterior hippocampal area has preferential connectivity to conceptual cognitive systems in the anterior neocortex. The anterior hippocampal neocortical connections support global representations that constitute the essence of memory (i.,e. gist), and the posterior hippocampal neocortical connections support detailed, perceptually rich memories. The anterior hippocampal section is connected to the medial prefrontal cortex (an important anterior neocortical structure for memory formation) via the entorhinal cortex. The entorhinal cortex provides information for the translation between gist and schemas (i.e., abstract mental representations that influence perceptual and memory processes). The medial prefrontal cortex supports the presentation of schemas for common aspects of memories across different episodes. In summary, long-term memory formation and transformation are enabled by the posterior-anterior hippocampal connectivity specialisation, the medial prefrontal cortex, and the entorhinal cortex, which support the representational gradient from fine to coarse and from perceptual to conceptual.

The MTT postulates that:

- episodic memory is dependent on the reactivation of hippocampal memory traces;
- semantic memories are primarily dependent on the neocortex, except for personally relevant semantic memories, which also depend on hippocampal connections through the medial temporal lobe; and
- memories change over time. Details are lost; the memory representation shifts over time from the posterior to the anterior hippocampal section, where the gist of memory is retained, and later to the medial prefrontal cortex and related structures in the anterior neocortex, where only the memory schemas remain without any hippocampal memory engagement [295,296,299].

Several critical predictions of the MTT have yet to be proved [300]. However, MTT provided a framework for developing new animal and clinical experimental protocols and alternative memory consolidation theories to understand, in more detail, the memory consolidation process in the human [300].

A recent alternative to the SCTs and MTT is the memory manifold theory (MMT). The MMT’s central premise is that no single variable can predict hippocampal input on memory formation, consolidation, and retention over time. The hippocampal contribution to memory is the product of interactions between multiple variables, including the amount of damaged or inactivated hippocampal regions, the amount of information transmitted by the hippocampus to distributed cortical areas to support the representation of schemas for common aspects of memories across different episodes, and the ability to compare stimulus information with information retrieved from memory by pattern separation, completion and matching cognitive activities [300].

### 5.3. Hippocampal Activity, Cognitive Tasks and Large-Scale Brain Networks’ Functional Connectivity in Healthy Adults

The hippocampus is central in cognition-related brain networks through its bilateral connections with anterior and posterior cortical areas. Anatomically discrete hippocampal areas in the medial to lateral direction are the subiculum, CA1–CA3 areas, and dentate gyrus, with segregated pathways to the parahippocampal areas. The development of high-resolution MRI has enabled the verification of animal and human brain measurements suggesting an anterior-posterior hippocampal memory function specialisation. Wael et al. integrated human brain MRI high-resolution structural and resting-state functional neuroimaging. They identified hippocampal segmentation along the anterior/posteriors and medial/ lateral directions in both hemispheres of healthy adults. Memory and emotional reactivity domains were identified in anterior hippocampal segments [297].

Additional evidence for the association of anatomically discrete hippocampal sections with specific large-scale brain networks in the brains of healthy adult participants was provided by Zheng et al. The authors presented experimental evidence for preferential anterior (i.e., head and body) hippocampal functional connections to the default mode network, and for the preferential posterior (i.e., tail) hippocampal functional connections to the goal-oriented and stimulus-recognition-supporting parietal memory network. Therefore, the hippocampus and medial parietal cortex are connected by two adjacent, functionally discrete and specialised brain networks, one for self-oriented and the other for goal-oriented processing [298].

### 5.4. Hippocampal Functional and Structural Connectivity within Large-Scale Brain Networks in Persons with SCI, MCI or AD

Grieder et al. examined the default mode’s changes in functional connectivity and multi-scale entropy of 15 patients in the early clinical stages of AD. Multi-scale entropy measures detect changes in the blood-oxygen-dependent signal within an area, presumed to precede changes in the default mode network connectivity between these brain areas. Compared to controls, patients in the early clinical stages of AD had reduced functional connectivity between the posterior cingulate cortex and right hippocampus, and no significant changes in the whole-network functional connectivity. However, the global multi-scale entropy value was lower than in the age-matched controls. This difference was most pronounced in the left and right hippocampi and consistent with the patients’ mini-mental state examination scores. The authors suggest that multi-scale entropy could be used as a supplemental marker for cognitive decline in conjunction with functional connectivity [301].

Disrupted functional connectivity in the SN brain regions (i.e., in the right anterior insula, left anterior insula, and anterior cingulate cortex) was observed in patients with MCI. A recent study quantified SN’s functional connectivity changes, hippocampal atrophy, and AD molecular markers with PET tracers for β-amyloid in a group of 33 age and sex-matched patients with MCI, and in 27 healthy controls. Compared to controls, the SN brain regions in patients with MCI had significantly more atrophy with an increased brain β-amyloid. These results were associated with lower mini-mental state examination scores [145].

MRI brain imaging of patients with aMCI consistently shows hippocampal, entorhinal, amygdalas’ and parahippocampal atrophy (collective parts of the medial temporal lobe). Agosta et al. evaluated sensorimotor network rewiring in 15 aMCI and 10 AD persons compared to 11 controls; all participants were right-handed. The aim was to test the hypothesis of early temporal medial lobe hyperactivation in aMCI, followed by late hypoactivation with the progression from aMCI to clinical AD. Functional brain network activity during the simple motor task was evaluated with fMRI and structural MRI to detect microstructural white matter brain changes. MCI patients had, compared to controls, reduced activity in the left inferior frontal gyrus. When compared with AD patients, aMCI participants had increased activity in the left postcentral gyrus and head of the left caudate nucleus, and decreased activation in the cingulum. The structural connectivity between the primary sensorimotor cortices of aMCI patients was changed compared to controls, based on diffusion-weighted brain images. Microstructural white matter brain changes were more widespread in patients with AD than in aMCI patients. They included structural connectivity changes between the left SMC, the head of the left caudate nucleus, and the cingulum. Changes in the functional and structural brain connectivity of aMCI patients were associated with hippocampal atrophy; in patients with AD, the altered brain activity correlated with widespread grey matter damage. The progressive changes in functional and structural brain connectivity during the transition from aMCI to clinical AD were attributed to the concomitant processes of brain networks’ rewiring and advanced structural damage [64].

Functional connectivity remapping of the posterior and anterior components of the DMN, the cerebellar network, and the left and right frontoparietal executive control network was studied in humans during the transition from normal cognition to MCI. Participants were evaluated with the letter fluency test, on which patients with MCI often achieve a score equal to normal cognitive persons. The study included 45 persons with MCI and an equal number of healthy controls that were age, sex and education matched. In addition to the letter fluency test, all participants completed a battery of cognitive tests, structural MRI and resting state fMRI. The letter fluency test was the only test where no differences were recorded between the two groups. Compared to MCI participants, healthy controls had higher functional connectivity within the posterior DMN and lower functional connectivity in the cerebellar and right frontoparietal executive control networks. Additionally, there were functional connectivity differences within the group of MCI participants when sub-divided into a high or a low-scoring group according to their overall cognitive test performances. Compared to low-scoring MCI participants, high-scoring MCI participants had increased functional connectivity within the anterior DMN; the bilateral executive control networks and all three networks had increased functional connectivity with the ventral and dorsal part of the Brodmann area 19, part of the occipital lobe cortex. Therefore, the cognitive abilities underpinning the normal test performance of MCI participants on the letter fluency test are supported by the occipital lobe’s long-range cortical connectivity [302].

He et al. implemented a longitudinal MRI study to compare the fractional anisotropy and mean diffusion rate of brain regions’ white matter in two groups of aMCI patients (who progressed to AD or remained at the level of aMCI) with the brain white matter integrity in normal controls. Compared to controls, both groups of aMCI patients had significantly reduced fractional anisotropy and increased diffusivity values in the cingulum, fornix, hippocampus, and uncinate fasciculus; changes were the greatest in aMCI patients who developed clinical signs of AD. The extent of microstructural white matter brain changes correlated with MCI patients’ mini-mental state examination scores. This correlation (most pronounced for the right cingulate and hippocampus) enabled the researchers to predict the probability of the MCI patient’s transition to clinical AD with 90% accuracy [63].

Shao et al. evaluated brain white matter integrity with MRI diffusion tensor tractography measurements in 22 persons with normal cognition, 22 patients with SCI and 25 with aMCI, and a neuropsychological assessment of all study participants. Compared to controls, participants with aMCI had widespread disruption of white matter tracts in the left anterior thalamic radiation, right corticospinal tract and left hippocampal cingulum; the SCI group had white matter tract disruption in the left hippocampal cingulum only. Therefore, the diffusion tensor tractography measurements in the SCI group were borderline white matter disruption between the normal control and aMCI groups. In the aMCI group, the disruption of white matter tracts was reflected in degraded cognition; the left hippocampal cingulum’s mean diffusivity was negatively correlated with the quality of episodic memory, and the radial diffusivity of the right corticospinal tract was negatively correlated with the quality of executive function. No statistically significant correlation was observed between the alterations of white matter integrity of the left hippocampal cingulum and the neuropsychological assessment scores in the SCI group [65].

A longitudinal study evaluated, at baseline and one year later, 124 persons (with an average age of 64 years) with SCI and a family history of AD using neuropsychological tests (for immediate and delayed memory, attention, language, and visuospatial functioning) and for functional connectivity between (a) the posterior DMN, anterior ventral DMN and medial temporal memory system, and (b) the connectivity of each these networks with the rest of the brain. A higher connectivity correlation value between the medial temporal memory system and the rest of the brain networks was positively correlated with better baseline immediate memory, attention, and global cognition. Over time, the quality of immediate memory was negatively correlated with higher correlation values for internal connectivity in the medial temporal memory system, and the connectivity between the posterior DMN and the medial temporal memory system. The authors concluded that persons with SCI have a brain connectivity pattern consistent with the early stage of AD-related brain network connectivity disruption, associated with diminished memory practice effects [303].

Patients with AD (n = 44), aMCI (n = 34) and 41 age- and sex-matched normal controls were evaluated with neuropsychological tests, fMRI for hippocampal functional connectivity with all brain regions, and MRI of fornix white matter microstructural integrity. Patients with AD or aMCI had decreased hippocampal volume, reduced functional connectivity between the left and right hippocampi and between the hippocampi and brain regions of the DMN and CN, and altered microstructural integrity of the fornix. Cognitive decline (detected with neuropsychological testing) was correlated with reduced left hippocampal connectivity and altered microstructural integrity (measured as decreased fractional anisotropy and increased diffusivity) of the fornix body. The authors concluded that hippocampal structural and functional dysconnectivity contributed to cognitive decline in patients with aMCI or AD [304].

Human cortical and subcortical brain regions, where hippocampal connections and large-scale brain network regions overlap, are summarised in Table 1.

## 6. Discussion and Conclusions

Decades of research on animal models of AD and post-mortem analysis of human brains of patients with clinically diagnosed AD have firmly established the high probability temporal association between cognitive decline (measured by attenuated memory formation, spatial learning, and object recognition) and perturbed brain Aβ and tau proteostasis. AD pathology, characterised by biochemical, histological, and electrophysiological techniques, was consistently observed first in the hippocampus and later included other neocortical brain regions with hippocampal connections [17,18,73,74,76,77,78,79,80,81,82,83,84,85,86,87,88,89,90,91,92,93,94,96,97,98,305]. However, there is no statistically significant difference in toxic AβOs levels between early and late AD [94], and high CSF soluble Aβ42 levels are associated with normal cognition, an average hippocampal volume, and PET-measured advanced brain amyloidosis [72].

High-resolution electron microscopy (EM) and immunohistochemistry (IHT) analysis of brain samples detected decreased spine density, dendritic spine morphological changes and an overall decreased hippocampal density of chemical synapses as an early sign of AD-related neuronal brain damage, preceding the neuronal loss comprehensively reviewed by Serrano et al. [11]. Neither electrical synapses with specific adhesion, scaffolding, and regulatory synaptic molecules [306,307] nor exocytosis regulating presynaptic proteins in astrocytes and microglia [11,184,185] have been morphologically quantified with IHT to the same degree as brain chemical synapses. EM and IHT techniques localised AD pathology’s early, presymptomatic changes to hippocampal chemical synapses. However, these techniques are unsuitable for the longitudinal evaluation of AD pathology in the living human brain.

Advances in structural and functional connectivity imaging techniques, developed during studies on the brains of living animal models and patients, have enabled the transition from clinical signs and symptoms-based AD diagnosis to a biomarkers-based diagnosis. This transition has promoted a multimodal approach to the study of AD that has increased the prediction of AD incidence and staging by combining the results of high-resolution structural and volumetric MRI, diffusion tensor imaging and fibre tractography MRI, fMRI, FDG-PET, PET imaging with radiotracers for brain synaptic density quantification, quantitative EEG, and body fluid sampling for synaptic protein markers, soluble Aβ peptides and tau [11,17,20,66,199,200,201,202,204,205,223,234,236,238,239,240,241,250,251,252,253,254,255].

The regional whole-brain synaptic density and corresponding synaptic function measurements do not overlap completely. Synaptic density markers are more resistant to the experimental protocol-elicited variables, and synaptic function markers are more likely to detect changes in the overall and regional differences in brain electrical activity, glucose concentration and blood oxygenation concentration [11]. Current in vivo brain PET synaptic density markers do not evaluate for synaptic density of electrical synapses. In contrast, in vivo, functional connectivity methods (e.g., fMRI) record the combined contribution of chemical and electrical synapses on large-scale brain networks.

In vivo brain imaging techniques have a significantly lower spatial resolution than EM or IHT techniques and can only evaluate synaptic density indirectly. However, brain imaging techniques for living patients compensate for this drawback by enabling longitudinal studies of whole-brain structural and functional connectivity by overlaying both connectivity studies. For example, high-resolution fMRI structural and resting-state functional neuroimaging identified hippocampal segmentation along the anterior/posteriors and medial/ lateral directions, with segregated pathways to the parahippocampal areas in both hemispheres of healthy adults. Memory and emotional reactivity domains were identified in anterior hippocampal segments [297]. Functional connectivity remapping of the posterior and anterior components of the DMN, the cerebellar network, and the left and right frontoparietal executive control network was detected in humans during the transition from normal cognition to MCI through analysis of structural MRI and resting state fMRI data [302].

Normal hippocampal function and connectivity are essential for semantic and episodic memory processing. Normal memory is critical for the normal operation of many cognitive subdomains such as learning, planning, prioritising, making decisions, responding to environments, moving between tasks, controlling personal desires, expressing empathy, recognising social cues, and self-motivation [289,292,295,296,297,298,299,300]. The central role of the hippocampus is further highlighted by the extensive and spatially segregated extrahippocampal connections with multiple cortical regions, including the perirhinal and the entorhinal cortex, parahippocampal cortex, association regions in the temporal and parietal lobes, and prefrontal cortex. Therefore, the consequences of AD-associated decreased hippocampal synaptic density or decreased hippocampal synaptic function are not localised to the hippocampus; they are amplified and reflected in the altered functional connectivity of intrinsic brain networks (aka large-scale networks) including the frontoparietal executive control, default mode, and salience networks [145,297,298,301,302].

Compared to normal controls, persons with SCI or MCI have measurable changes in hippocampal functional and structural connectivity within large-scale brain networks. These changes are reflected in lower cognitive test scores [63,64,65,145,302,303,304]. Therefore, brain functional and structural connectivity imaging in living patients can detect early cognitive changes associated with pre-clinical AD: SCI and MCI. For example, fMRI brain measurements can measure the functional connectivity remapping of large-scale brain networks in persons with MCI [302], and longitudinal MRI can be used to evaluate brain white matter integrity and predict the patient’s transition from MCI to clinical AD [63].

In conclusion, there is a need to measure the contribution of (a) electrical synapses and (b) presynaptic proteins released by astrocytes and microglia to AD pathology. This additional knowledge would improve the prediction and early diagnosis of AD in humans. Therefore, there is a need to develop specific biomarkers for in vivo brain imaging of electrical synapses and for imaging astrocyte and microglia-associated molecules that modulate exocytosis in chemical synapses.

## Figures and Tables

**Table 1:** Human cortical and subcortical brain regions with overlapping between hippocampal and large-scale brain networks’ connections.

Large Scale Network	Default Mode	Salience	Dorsal Frontoparietal	Lateral Frontoparietal (Central Executive)
Cortical or Subcortical Regions				
Amygdala	●			
Cingulate cortex		●		●
Parietal cortex				●
Prefrontal cortex	●		●	●
Temporal cortex				●
Thalamus	●			

## Data Availability

No data available.

## Abbreviations

[11C]PiB	(11)C-labeled Pittsburgh Compound-B
[11C]UCBJ	(4R)-1-[(3-(11C)Methylpyridin-4-yl)methyl]-4-(3,4,5-trifluorophenyl)pyrrolidin-2-one
[18F]	fluorine radioisotope
[18F]FDG	[18F]Fluorodeoxyglucose
[18F]MNI-1126	18F analog of [11C]UCB-J PET radioligand
[18F]SDM-16	(4R)-4-(3-fluoro-5-(fluoro-18F)phenyl)-1-((2-methyl-1H-imidazol-1-yl)methyl)pyrrolidin-2-one
[18F]SDM-16	(4R)-4-(3-(18F)fluoranyl-5-fluorophenyl)-1-[(3-methylpyridin-4-yl)methyl]pyrrolidin-2-one (UIPAC name)
[18F]SDM-8	(4R)-4-(3-(18F)fluoranyl-5-fluorophenyl)-1-[(3-methylpyridin-4-yl)methyl]pyrrolidin-2-one
[18F]SynVesT-1	(4R)-4-(3-(18F)fluoranyl-5-fluorophenyl)-1-[(3-methylpyridin-4-yl)methyl]pyrrolidin-2-one
[18F]UCB-H	(4R)-1-((3-Fluoranyl-4-pyridyl)methyl)-4-(3,4,5-trifluorophenyl)pyrrolidin-2-one
99-CTF	99-amino acid membrane bound C-terminal fragment
99-CTF	99-amino acid membrane-bound C-terminal fragment
Aβ	amyloid β
AβF	amyloid β fibril
AβPF	amyloid β protofibril
AD	Alzheimer’s disease
ADHD	attention deficit hyperactivity disorder
aMCI	amnestic mild cognitive impairment
AMPA	α-amino-3-hydroxy-5-methyl-4-isoxazolepropionic acid
AMPAR	α-amino-3-hydroxy-5-methyl-4-isoxazolepropionic acid receptor
APOE3	apolipoprotein E3
APOE4	apolipoprotein E4
APP	amyloid-β precursor protein
APP	amyloid precursor protein
APP669–711	amyloid-β precursor protein isoforms
ATN	(amyloidβ (A), phosphorylated tau (T) and neurodegeneration (N) markers)
ATP	adenosine triphosphate
AβO(s)	amyloid β oligomer(s)
BDNF	brain-derived neurotrophic factor
BOLD	blood oxygenation level-dependent
bvFTD	behavioural variant of frontotemporal dementia
CA1	hippocampal subfield/region CA1
CaMKII	Ca2+/calmodulin-dependent protein kinase II
cdk5	cyclin-dependent kinase 5
CJD	Creutzfeldt–Jakob disease
CREB	cAMP response element-binding protein
CSF	cerebrospinal fluid
DAB1	DAB adaptor protein 1
DAB1-P	phosphorylated DAB adaptor protein 1
DMN	default mode network
DNA	deoxyribonucleic acid
DTI	diffusion tensor imaging
EEG	electroencephalogram
EM	electron microscopy
EMG	electromyography
EphB2	ephrin type-B receptor 2
FAD	familial Alzheimer’s disease
FDG-PET	fluorine-18 fluorodeoxyglucose PET imaging
fMRI	functional magnetic resonance imaging
FYN	proto-oncogene tyrosine-protein kinase Fyn
GABA	gamma-aminobutyric acid
GABAB	gamma-aminobutyric acid B receptor
GAP-43	growth-associated protein 43
GluA2	ligand-gated ion channel glutamate receptor
GluCEST	glutamate chemical exchange saturation transfer
GluN1	NMDA receptor subunit GluN1
GluN2B	NMDA receptor subunit GluN2B
GSK3β	glycogen synthase kinase-3β
HT	histochemical techniques
IDE	insulin-degrading enzyme
IFITM	interferon-induced transmembrane protein
IHC	immunohistochemistry
IHT	immunohistochemical techniques
LilrB2	leukocyte immunoglobulin-like receptor subfamily B member 2
LOAD	late-onset form
LTD	long-term depression
LTP	long-term potentiation
M1 microglia	microglia releasing inflammatory mediators, promotes inflammation and neurotoxicity
M2 microglia	microglia releasing anti-inflammatory mediators, has an anti-inflammatory and neuroprotective effect.
MCI	mild cognitive impairment
MEG	magnetoencephalography
mGluR5	metabotropic glutamate receptor 5
miRs	microRNAs
MMT	memory manifold theory
MMT	memory manifold theory
MRS	MR spectroscopy
MT	mitochondria(l)
MTT	multiple trace theory
NCAM	neural cell adhesion molecule
NFκB	nuclear factor kappa-light-chain-enhancer of activated B cells
NfL	neurofilament light polypeptide/chain
NFT	neurofibrillary tangles
NgR1	negative growth regulatory protein NGR1
NMDAR	N-methyl-D-aspartate receptor, tetrameric protein with two GluN1 subunits and two GluN2 (A-D) subunits; a type of L-glutamate receptor
NPT2	sodium-dependent phosphate transport protein 2A
NS	nucleation site
P-tau	phosphorylated tau protein
PET	positron emission tomography
PET SV2	PET synaptic vesicle protein 2A tracer
PI3K	phosphoinositide 3-kinase
PirB	paired immunoglobulin-like receptor
PKA	protein kinase A
PKB	protein kinase B
PMN	parietal memory network
PSD-95	postsynaptic density protein 95 (a postsynaptic scaffolding protein in excitatory neurons)
PSD(s)	postsynaptic density protein(s)
R-fMRI	resting state fMRI, evaluates intrinsic brain networks at rest by calculating correlations between brain regions
Rab	member of the Ras superfamily small G proteins
RAGE	receptor for advanced glycation end products, aka AGER
ROS	reactive oxygen species
SCI	subjective cognitive impairment
SCTs	standard consolidation theories
SEM	scanning electron microscopy
SMC	sensorimotor cortex
SNAP-25	synaptosomal-associated protein 25
SPECT	single-photon emission computerized tomography
SV2(A)	synaptic vesicle glycoprotein 2(A)
T-tau	total tau
TEM	transmission electron microscopy
TNFα	tumour necrosis factor α
VaD	vascular dementia
VDAC1	voltage-dependent anion-selective channel 1
